# Integrated Size-Selective Cell Purification and Electroporation for Genetic Manipulation of Primary Cells

**DOI:** 10.3390/mi17030359

**Published:** 2026-03-15

**Authors:** Hyun Woo Sung, Soojung Claire Hur

**Affiliations:** 1 Department of Chemical and Biomolecular Engineering, Johns Hopkins University, 3400 N Charles Street, Baltimore, MD 21218, USA; hsung10@jhu.edu; 2 Department of Mechanical Engineering, Johns Hopkins University, 3400 N Charles Street, Baltimore, MD 21218, USA; 3 Institute of NanoBioTechnology, Johns Hopkins University, 3400 N Charles Street, Baltimore, MD 21218, USA; 4 The Sidney Kimmel Comprehensive Cancer Center, Johns Hopkins Hospital, 401 N Broadway, Baltimore, MD 21287, USA

**Keywords:** microfluidic electroporation, size-based cell selection, primary cell transfection, integrated cell processing

## Abstract

Biologically relevant primary cell samples are inherently heterogeneous and often require selective enrichment prior to genetic manipulation. We previously demonstrated a vortex-assisted microfluidic platform that integrates size-selective cell trapping with electroporation; however, its limited processing capacity constrained applications requiring larger sample volumes. Here, we present a scaled version of this integrated system achieved through electrode array redesign and electrical optimization. The updated architecture increases processing capacity while preserving size-selective trapping behavior, electric field uniformity, and device stability. Systematic optimization of electrical and buffer conditions enables efficient delivery of plasmid DNA and in vitro-transcribed mRNA into primary human cells, with performance approaching benchmark chemical transfection methods. By scaling an integrated trapping–electroporation workflow without compromising delivery performance, this platform advances microfluidic cell engineering toward practical processing of heterogeneous primary cell samples.

## 1. Introduction

Biologically relevant cell samples, particularly those derived from patient tissues or biofluids, are inherently heterogeneous and often contain subpopulations that require selective enrichment prior to functional analysis or genetic manipulation. While primary human cells offer physiologically meaningful models, their limited proliferative capacity [1,2] and increased susceptibility to membrane damage and viability loss during *in vitro* manipulation [3,4,5,6] make intracellular delivery of genetic material especially challenging.

Chemical and viral gene delivery approaches introduce additional limitations, including reliance on carrier materials, cytotoxicity, immunogenicity, potential genome integration, and complex pre-selection workflows [7]. As alternatives, physical intracellular delivery strategies have been developed to facilitate membrane permeabilization without exogenous carriers.

Mechanically assisted intracellular delivery approaches, including nanoneedle-based platforms [8], cell-squeezing approaches [9], and recent high-throughput 2D mechanoporation systems [10], induce membrane disruption through localized deformation. These systems have demonstrated efficient intracellular delivery with preserved cell viability. However, challenges related to device fabrication complexity, substrate dependence, and integration within heterogeneous sample-processing workflows remain active areas of investigation.

Electroporation represents another widely adopted physical delivery strategy, enabling transient membrane permeabilization through externally applied electric fields. Performance can be tuned through electric field strength, pulse duration, and buffer composition [4,5,11,12,13,14]. Nevertheless, conventional bulk electroporation systems often require high voltages and may generate spatially non-uniform electric fields, resulting in variable membrane permeabilization and reduced reproducibility, particularly when applied to heterogeneous primary cell populations [14,15].

Microscale electroporation platforms implemented within microfluidic architectures have addressed several of these limitations by enabling localized field control, precise fluid handling, and parallelized operation [16,17,18,19]. Designs including microelectrode arrays [20], continuous flow-through electroporation systems [18,19,21,22], and droplet-based formats [23,24], have demonstrated efficient gene delivery to primary cells. However, most focus exclusively on intracellular delivery and assume pre-purified, tightly size-distributed input populations, leaving upstream cell isolation and downstream electroporation as separate processes.

In practice, workflows involving heterogeneous samples therefore require sequential cell isolation, buffer exchange, and electroporation steps, introducing handling losses and limiting overall efficiency. Integrating selective cell processing with controlled intracellular delivery within a unified microfluidic platform remains a key engineering challenge for translating microscale electroporation technologies toward practical applications.

Building on this need for workflow integration, we previously developed a vortex-assisted microfluidic platform that combines vortex-based cell trapping with tunable electroporation [25,26,27,28]. Vortex trapping geometry has been independently validated for enrichment of rare human cells from complex biofluids [29,30,31], and its coupling with electroporation established the feasibility for multi-molecular delivery [25,26,27] and application to model heterogeneous samples using a spike-in cancer cell system [28]. However, the limited processing capacity of the earlier prototypes constrained operation at larger sample volumes and restricted validation primarily to immortalized cell lines and controlled model systems.

**Figure 1:**
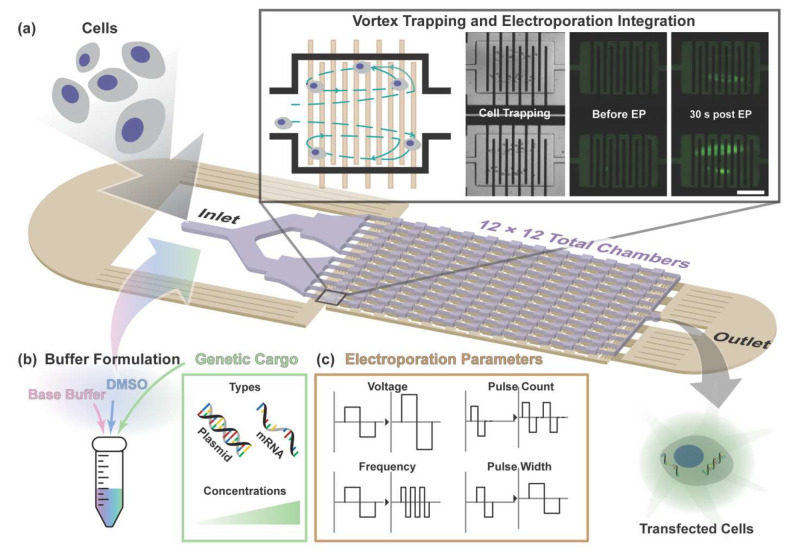
System overview. (**a**) Schematic of the vortex-assisted electroporation platform with enhanced processing capacity. Cells enter through the inlet and are hydrodynamically trapped within microscale vortex chambers in a size-selective manner, retaining cells above a designed diameter threshold for electroporation using an integrated electrode array. Representative brightfield and fluorescence images illustrate cell trapping and electroporation before and after pulse application. Following electroporation, transfected cells are collected at the outlet. (**b**) Buffer formulation and cargo modularity. The electroporation buffer is assembled from commonly used laboratory reagents, including a base buffer supplemented with DMSO and genetic cargo. This formulation strategy provides flexibility in selecting and adjusting buffer compositions across different types, while supporting delivery of diverse cargo types, including plasmid DNA and mRNA, and enabling performance tuning through cargo concentration. (**c**) Electroporation parameter control. Voltage amplitude, pulse count, pulse frequency, and pulse width are independently tunable within the platform, enabling precise control of electrical stimulation during electroporation.

Here, we extend this integrated platform through redesign of the electrode array and electrical routing to increase processing capacity while preserving size-selective trapping behavior and electric field control (Figure 1). Rather than altering the fundamental trapping mechanism, which has been commercially validated for rare-cell enrichment in complex biofluids [29,30,31], the updated design focuses on improving electrical efficiency and operational stability to enable higher-volume processing within the same integrated workflow. Using this enhanced platform, we systematically examine electroporation parameters, including buffer composition, electric field strength, and cargo concentration, in primary human cells. Platform versatility is further evaluated through delivery of both plasmid DNA and in vitro-transcribed (IVT) mRNA, enabling assessment across cargos of differing molecular size and expression dynamics.

## 2. Methods

### 2.1. Device Fabrication

The device consists of a polydimethylsiloxane (PDMS) layer with the vortex cell-trapping chambers [27,29] enclosed by a glass slide with a microfabricated gold electrode array patterned on its surface. The microfluidic channel height was 75 µm, as defined by the photoresist mold (KMPR 1050, MicroChem, Westborogh, MA, USA) used during soft lithography. Detailed fabrication procedures are described in our previous report [28]. In brief, the device consists of a PDMS layer (cat# 4019862, Dow, Midland, MI, USA) plasma-bonded to a micropatterned Au-electrode glass slide (cat# 48300-026, Avantor, Radnor, PA, USA). The PDMS layer is a microfluidic chip comprising an inlet accommodating multiple solution ports, a microchannel network, and an outlet. To prevent debris in biological fluids from potentially blocking the microchannels, coarse filters were imprinted prior to the microchannels. The device has a single outlet port for washing buffer waste removal, sample cycle recycling, and collection. Inlet and outlet ports were punched out using a 20-gauge blunt tip needle (cat# 90120050D, CML Supply, Lexington, KY, USA). The PDMS chip was manually aligned to the electrode array under stereo microscopy after the plasma treatment step (Reactive Ion Etch Series 800 Plasma System, MICRO-RIE, Technics Inc., Naperville, IL USA).

### 2.2. Electrode Design and Modeling

We adopted an electrode design and modeling approach, building upon previously established methodologies [27], with key modifications to enhance processing capacity while preserving electroporation performance. Our focus was to develop a new electrode arrangement that enhances sample processing capacity while maintaining per-chamber, vortex-assisted electroporation performance comparable to our previous low-throughput design. To enable uniform voltage distribution and synchronized operation across all chambers, the chamber electrodes were connected in a series-parallel configuration, balancing the electrical load while preserving efficient electroporation conditions. We evaluated the patterned Au electrodes by converting them into equivalent circuit diagrams (Figure S3). Each segment of the patterned Au was approximated as a rectangular section with a uniform cross-section and material composition, and the segment resistance, *R*, was computed from resistivity, ρ, as:

(1) $$R=\rho \frac{l}{A}=\rho \frac{l}{wh\;}$$

Here, ρ is the resistivity of Au (2.44 × 10^−8^ Ωm for our calculations), l is the segment length, and *A* is the cross-sectional area defined by the width, *w*, and height, *h*. The electrode thickness, *t*, was fixed at 300 nm to ensure that stable plasma bonding between the PDMS and the glass slide could occur. Each chamber used five pairs of interdigitated electrodes (450 µm length and 20 µm width) connected in opposite polarity via a common bus line (Figure S3b). The electrical resistance of the per-chamber electrode network was estimated to be 401.6 Ω using COMSOL Multiphysics (version 5.2, Burlington, MA, USA) simulations with DPBS as the cell suspending medium. The final electrode schematic, along with resistance estimates, was translated onto SPICE models to simulate the voltage drop across each electrode (Figure S3b).

Because the interdigitated electrode geometry within each chamber was unchanged from our previously reported 4 × 10 design [27], local electric field distributions within individual electroporation chambers were assumed to be consistent with prior COMSOL Multiphysics simulations. In that work, spatial electric field profiles were computed under applied voltage conditions using DPBS as the suspending medium, and the mean electric field was determined based on the inter-electrode spacing and applied potential difference. Reported electric field intensities were extracted at a height of Z = 60 µm from the base of the electrodes, corresponding to the cell-trapping vortex ring located farthest from the electrode surface within the 75 µm-high microfluidic channel, thereby providing a representative field estimate at the cell recirculating region rather than at the electrode interface. In the present study, Simulation PRogram with Integrated Circuit Emphasis (SPICE) modeling was used to determine voltage distribution across the expanded array, after which the effective electric field within each chamber was estimated from the simulated per-chamber voltage drop divided by the electrode gap distance, providing an average field approximation within the electrode gap region. This approach decouples global routing resistance effects from local electric field geometry.

### 2.3. Cell Culture

Immortalized cell lines (MCF-7 and HEK 293 cells) were purchased from ATCC (Manassas, VA, USA) and maintained in Dulbecco’s Modified Eagle Medium (DMEM) (cat# 11995065, Thermo Fisher, Waltham, MA, USA) supplemented with 10% (*v*/*v*) heat-inactivated fetal bovine serum (HI-FBS) (cat# 16140071, Thermo Fisher, Waltham, MA, USA) and 1% (*v*/*v*) penicillin-streptomycin (cat# 15140122, Thermo Fisher, Waltham, MA, USA). Cells were passaged at 70% confluency.

HMFs (cat# 7630, ScienCell, Carlsbad, CA, USA) were maintained following the manufacturer’s protocol at 37 °C in a 5% CO_2_-humidified incubator. Cells were passaged at 90% confluency. PD was calculated by taking the base-2 log of the ratio between the total cells collected before subculture and total cells split into a new vessel. Starting from a PD of 0 at passage 1, the PD of low- and high-passage cells were between 7–14 and 14–30, respectively. Unless otherwise stated, HMF cells with PD < 14 were utilized for all plasmid and mRNA electroporation experiments.

All cells used in our experiments were subcultured using 0.05% (*v*/*v*) of Trypsin-EDTA (cat# 25200056, Thermo Fisher, Waltham, MA, USA) diluted in DPBS following standard protocol.

### 2.4. Electroporation Buffer Preparation

DPBS (cat# 14190144, Thermo Fisher, Waltham, MA, USA) and Opti-MEM^TM^ (cat# 51985034, Thermo Fisher, Waltham, MA, USA) were utilized as a base electroporation (EP) buffer for this study. Plasmids and mRNA were directly diluted into the base EP buffer. For experiments that added dimethyl sulfoxide (DMSO) to the EP buffer, 1% (*v*/*v*) DMSO (cat#45001-118, Corning, Corning, NY, USA) was added to each electroporation buffer directly prior to electroporation to minimize base pairing interaction with the genetic cargo.

### 2.5. Solution Exchange System

The positive-pressure pneumatic flow control system described in our previous works was utilized for solution exchange in this study [26,27,28]. To interface this system with standard labware, we 3D-printed a tube holder that couples the pneumatic line to standard 50 mL Falcon tubes (cat# 21008-178, Avantor, Radnor, PA, USA), enabling the controlled injection of solutions into the microfluidic devices. To assess cell-trapping efficiency, we connected two solution ports—one for the DPBS wash buffer and the other for the cell suspension—to the device’s inlet. For gene delivery experiments, three solution ports—DPBS with 1% (*v*/*v*) DMSO wash buffer, the customized electroporation buffer, and the cell suspension (cells resuspended in growth media)—were connected to the inlet of the device.

### 2.6. Trapping Efficiency Experimental Procedure

MCF-7 cells were trypsinized and pelleted by centrifugation at 1100 rpm for 5 min and diluted in fresh media to a final concentration of 5 × 10^3^ cells/mL. Cells were then stained with 1 µM Calcein AM (cat# 564061, BD Pharmingen, San Diego, CA, USA) for 10 min prior to cell trapping efficiency experiments. Next, 200 µL of this cell suspension was diluted with 40 mL of media (1 × 10^3^ cells, 25 cells/mL) in 50 mL syringes (cat# 13-689-8, Fisher Scientific, Waltham, MA, USA). For the cell trapping syringe sequence, formation of vortex trapping flow rates was first primed at 40 psi from the DPBS vial for 30 s. With the DPBS still flowing, a separate syringe pump (cat# 70-3007, Harvard Apparatus, Holliston, MA, USA) then infused the cell suspension at 5 mL/min for 50 s (total flow rate through device = 5.2 mL/min, *Re* = 136), followed by a withdrawal step at 0.5 mL/min for 30 s. This withdrawal step is necessary to ensure that only the vortex-trapped cells are collected during the collection procedure. After processing, cells were collected into 96-well plates (cat# 07-000-162, Fisher Scientific, Waltham, MA, USA) and imaged within 2 h. Cell diameters were measured from fluorescence images using an intensity-based image-recognition analysis, as described previously [28].

### 2.7. Membrane-Impermeable Molecule Electroporation Experimental Procedure

MCF-7 cells were trypsinized and pelleted by centrifugation (Thermo Centra CL2 Centrifuge, Thermo Fisher, Waltham, MA, USA) at 1100 rpm for 5 min and resuspended in fresh media to a final concentration of 1 × 10^3^ cells/mL. The cells were then stained with the nucleus dye NucBlue™ Live ReadyProbes™ (cat# R37605, Thermo Fisher, Waltham, MA, USA) according to the manufacturer’s protocol to label viable cells pre-electroporation. Then, 4 mL of this suspension was loaded into a 5 mL syringe and infused with the syringe pump at 4 mL/min for 50 s. After the cell trapping syringe sequence, the solution port was switched from the DPBS wash vial to the electroporation buffer vial. All membrane-impermeable molecule delivery experiments were conducted using DPBS as the base electroporation buffer. For YOYO-1 (cat# Y3601, Thermo Fisher, Waltham, MA, USA) delivery, electrical parameters were 10 pulses of 1 ms AC square waves at 10 kHz, with 1 s inter-pulse interval. Upon completion, cells were collected by reducing the pressure in the solution vials from 40 psi to 30 psi for 10 s, then transferred into 96-well plates at 5 psi for an additional 10 s. Immediately after collection, 100 µL of pre-warmed DPBS containing 2 µM Calcein Red-Orange, AM (cat# C34851, Thermo Fisher, Waltham, MA, USA, final well concentration ~1 µM) was added to each well, and the 96-well plate was placed in a humidified incubator at 37 °C with 5% CO_2_. At 20 min post-collection, the solution was replaced with 100 µL of pre-warmed DPBS, and wells were imaged using fluorescence microscopy.

### 2.8. Plasmid Preparation and Extraction

The plasmid pZsGreen1-C1 (hereafter referred to as ZsGreen) was obtained from Takara Bio (cat# 632447, Kusatsu, Shiga, Japan). The plasmid pcDNA-3xHA-hTERT was obtained from Addgene (ID: 51637, Watertown, MA, USA). The plasmid pcDNA3.1(+) eGFP was obtained from Addgene (ID: 129020, Watertown, MA, USA). Plasmids were transformed into *E. coli* DH5α cells (cat# 18265-017, Thermo Fisher, Waltham, MA, USA) for amplification, unless stated otherwise. Transformed cells were cultured in Luria–Bertani broth (cat# 12795027, Thermo Fisher, Waltham, MA, USA) prepared according to the manufacturer’s protocol. Glycerol stocks were prepared by mixing the inoculated broth 1:1 with 50% (*v*/*v*) sterile glycerol (cat#M153-100 ML, PANTek Technologies, Englishtown, NJ, USA) diluted in autoclaved water, and stored at −80 °C.

Glycerol stocks were used to inoculate 5 mL overnight starter culture at 37 °C with constant agitation (240 rpm). The starter culture was then expanded to a maxi-scale (>100 mL) culture grown for 12 h at 37 °C with reduced agitation (160 rpm) to minimize foaming. Plasmids were extracted using the Plasmid Plus Maxi Prep (cat# 12963, QIAGEN, Hilden, Germany) by following the manufacturer’s protocol. Plasmid concentration and purity were assessed by Nanodrop mode on a spectrophotometer (Take3 Micro-Volume Plate, BioTek Cytation 5, Winooski, VT, USA).

For quantitative comparison, microscale electroporation transfection efficiencies were normalized to those obtained using conventional transfection agents. Conventional agents were Lipofectamine™ 3000 (cat# L3000001, Thermo Fisher, Waltham, MA, USA) for plasmid transfection, and Lipofectamine™ MessengerMAX™ (cat# LMRNA001, Thermo Fisher, Waltham, MA, USA) for mRNA transfection. All transfections followed the manufacturers’ protocols. Fluorescence thresholds were set to the maximum signal emitted from non-electroporated cells.

### 2.9. In Vitro mRNA Synthesis

The plasmid pcDNA3.1(+) eGFP (hereafter referred to as eGFP) was obtained from Addgene (ID: 129020, Watertown, MA, USA). Glycerol stocks were used to inoculate 5 mL overnight starter cultures at 37 °C with constant agitation (240 rpm). The plasmid was then extracted using a spin miniprep kit (cat# 27104, QIAGEN, Hilden, Germany) following the manufacturer’s protocol and linearized by a single-site restriction enzyme digestion (BbsI-HF, cat# R3539S or PmeI, cat# R0560S, New England Biolabs, Ipswich, MA, USA) according to the manufacturer’s instructions. In vitro transcription (IVT) was carried out using T7 RNA polymerase (cat# M0251S, New England Biolabs, Ipswich, MA, USA) with either CleanCap Reagent AG (cat#N-7113, TriLink Biotechnologies, San Diego, CA, USA) or Anti-Reverse Cap Analog (ARCA) (cat# S1411S, New England Biolabs, Ipswich, MA, USA), substituting uridine entirely with N1-Methylpseudouridine-5′-Triphosphate (N1MePsU) (cat# N-1081, TriLink Biotechnologies, San Diego, CA, USA). The correct 5′-AG-3′ initiating sequence immediately following the T7 promoter site for CleanCap Reagent AG mRNA synthesis was introduced via site-directed mutagenesis using the Q5 Hot Start High-Fidelity DNA Polymerase Kit (cat# M0493S, New England Biolabs, Ipswich, MA, USA), with the following primers: forward: 5′- ACT CAC TAT AAG GAG ACC CAA G -3′ and reverse: 5′- CGT ATT AAT TTC GAT AAG CCA G -3′ (Integrated DNA Technologies). The mutation in the template was confirmed by Sanger sequencing. ARCA-capped mRNA was synthesized from the unmutated template. A Poly-(A) tail of approximately 100 nt was added using *E. coli* Poly(A) Polymerase (cat#M0276L, New England Biolabs, This page contains the following errors:) to the mRNA product. After each step, RNA products were purified using an RNA Cleanup Kit (cat# T2050S, New England Biolabs, Ipswich, MA, USA). RNA concentration and purity were assessed using a Nanodrop spectrophotometer. Commercially available eGFP mRNA with 5-methoxyuridine (5 moU) uridine substitution (cat# L-7201, TriLink Biotechnologies, San Diego, CA, USA) was purchased to compare the translation efficiency of in vitro transcribed mRNA products.

### 2.10. Electroporation-Mediated Gene Transfection Procedure

HEK293 and HMFs were trypsinized, pelleted by centrifugation at 1100 rpm for 5 min, and resuspended in fresh media to a final concentration of 1 × 10^3^ cells/mL. Then, 4 mL of this suspension was loaded into a 5 mL syringe (cat# 75846-756, Avantor Science, Radnor, PA, USA). The wash buffer was replaced from DPBS to DPBS with 1% DMSO (*v*/*v*) for all gene electroporation experiments. Following the syringe sequence described in previous sections (Section 2.6), cells were trapped in microvortices for an additional 2 min, then the active solution port was switched from the wash buffer to the EP buffer. Electroporation pulses were applied while maintaining an infusion pressure of 40 psi in the EP buffer. Unless otherwise stated, the electrical parameters for gene delivery were 20 pulses of 1 ms AC square waves at 10 kHz, with 1 s inter-pulse interval. Upon completion, cells were collected by reducing the vial pressure from 40 psi to 30 psi for 10 s, then transferring them into 96-well plates at 5 psi for an additional 10 s. Immediately after collection, 100 µL of pre-warmed medium was added to each well, and the 96-well plate was placed in a humidified incubator at 37 °C with 5% CO_2_. At 1 h post-collection, the medium was replaced with 100 µL of pre-warmed medium to completely remove residual electroporation buffer. Transfection outcomes were quantified by imaging at 24 h (mRNA) and 48 h (plasmid) post-electroporation to enumerate reporter-positive cells (e.g., GFP or HA), using direct reporter fluorescence for GFP and immunofluorescence for HA.

### 2.11. Immunofluorescence

All immunofluorescence experiments were carried out in 96-well plates unless stated otherwise. Cells were fixed with 4% paraformaldehyde (cat# 15710, Electron Microscopy Sciences, Hatfield, PA, USA) for 20 min and permeabilized with 0.1% Triton X-100 (cat# T8787, Sigma Aldrich, St. Louis, MO, USA) in DPBS for 2 min. Primary antibodies against HA (rabbit anti-HA C29F4 cat# 3724S, Cell Signaling Technology, Danvers, MA, USA, 1:200 dilution) or Ki67 (mouse anti-Ki67 8D5 cat# 9449S, Cell Signaling Technology, Danvers, MA, USA, 1:500 dilution) were diluted in 10% normal goat serum (cat# 50062Z, Thermo Fisher, Waltham, MA, USA) and incubated for 1 h at room temperature in a humidified chamber. Subsequently, cells were incubated with Alexa Fluor-conjugated secondary antibodies (anti-rabbit cat# 4414S, Cell Signaling Technology, Danvers, MA, USA, 1:3000 dilution) (anti-mouse cat# A-11001, Thermo Fisher, Waltham, MA, USA, 1:1000 dilution) and DAPI nuclei counterstain (0.2 µg/well; cat# D3571, Thermo Fisher, Waltham, MA, USA), both diluted in 10% normal goat serum, for 1 h at room temperature in a humidified chamber. Between each step, each well was washed three times with PBS-Tween (0.05% *v*/*v*; cat# 85113, Thermo Fisher, Waltham, MA, USA).

## 3. Results

### 3.1. Electrode Array Design for Scalable Integrated Operation

Enabling higher-volume operation within the integrated trapping–electroporation workflow while preserving field precision and consistency required redesign of the existing electrode array architecture that extends the prior 4 × 10 layout (4 parallel transverse rows, 10 longitudinal columns; 40 chambers) [27,28]. The updated array enables increased processing capacity while addressing critical engineering constraints, including chamber-to-chamber electric field uniformity and voltage drop across the inlet and outlet routing regions, which can otherwise compromise field uniformity in parallelized electroporation systems. The primary goal of the redesign was ensuring that the applied voltage translated efficiently and uniformly into consistent electric fields across all electroporation chambers —an essential requirement for reproducible gene delivery in a scaled integrated architecture.

Design efforts were shaped by multiple physical and fabrication constraints: (1) the maximum device footprint permitted by standard microscopic slides, (2) feature size selections made to ensure robust, high-yield photolithographic fabrication, (3) chamber spacing requirements necessary for fully developed vortex flow [29], and (4) electrode trace width and thickness requirements that preserve exposed glass for leak-proof PDMS-to-glass bonding. These constraints restricted the allowable array dimensions and necessitated careful optimization of the routing pathways within confined regions while maintaining compatibility with the established vortex trapping geometry [29].

**Figure 2:**
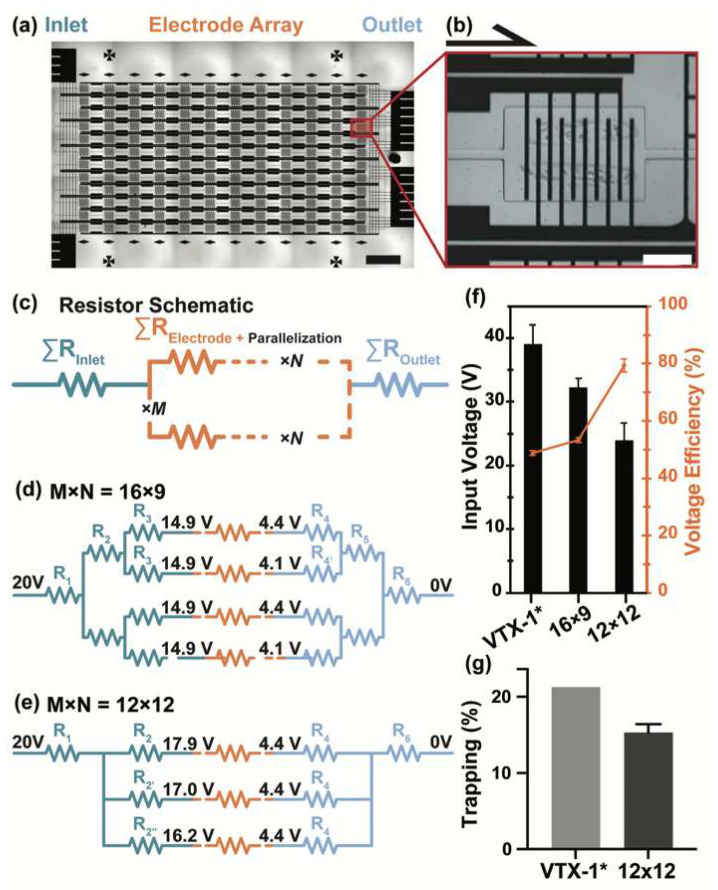
Design and electrical optimization of a scaled electroporation array. (**a**) Brightfield image of the electrode layout on the electroporation chip, consisting of an inlet (teal), an electroporation array (orange), and an outlet routing section (light blue). Scale bar = 2 mm. (**b**) Magnified brightfield image of a single electroporation chamber, showing cells trapped in vortices circulating around interdigitated electrodes. Scale bar = 250 µm. (**c**) Simplified resistance network representation of the electrode array, partitioned into inlet routing, electrode array, and outlet routing regions. Array parallelization (M × N) denotes the number of electroporation chambers generated through lateral (M) and longitudinal (N) replication of a base electrode unit. (**d**) Resistance network corresponding to the 16 × 9 layout, in which symmetric bifurcation of the inlet routing electrode produces two dominant resistance paths, reducing chamber-to-chamber variability. (**e**) Resistance network corresponding to the 12 × 12 layout, incorporating shortened routing paths and reduced inlet and outlet resistance, thereby shifting a greater fraction of the applied voltage drop onto the electrode array and improving voltage efficiency at the expense of increased resistive asymmetry. For all resistance networks shown, annotated voltage values (black) indicate simulated voltage drops between regions under a 20 V applied potential. Only the upper half of the network (M/2) is illustrated, as symmetry yields identical resistive behavior in both halves. (**f**) Input voltage required to achieve a mean electric field of 900 V/cm within the electrode array (left axis) and corresponding voltage efficiency (right axis). The 12 × 12 configuration requires substantially lower input voltage than the 16 × 9 and VTX-1 configurations, while exhibiting higher voltage efficiency. Error bars for input voltage indicate spatial voltage variability across chambers (standard deviation), whereas voltage efficiency error bars represent row-to-row variability. (**g**) First-cycle cell trapping efficiency comparison between the ultra-high throughput device (VTX-1; data referenced from refs. [29,30], purification-only platform) and the integrated 12 × 12 electroporation device. * Error bars represent mean ± SEM (*n* = 3).

Several candidate electrode array configurations were evaluated to balance processing capacity within the trapping chamber array and electric field uniformity. Each layout contained three main functional sections: an inlet routing pathway, the electrode array, and an outlet routing pathway (Figure 2a). Three array configurations—16 × 12 (VTX-1 style, 192 chambers) [29], 16 × 9 (144 chambers), and the final 12 × 12 (144 chambers)—were compared to assess the tradeoffs between field uniformity and electrical efficiency. Each chamber incorporated five pairs of interdigitated electrodes (IDEs) that generated localized electric fields for electroporating vortex-trapped cells (Figure 2b). Compared with planar parallel-plate configurations, the small inter-electrode gap in IDE geometries produces high local electric field strength at relatively low applied voltages. In the present device, vortex-trapped cells recirculate away from the electrode surface, occupying approximately the mid-to-upper portion of the microfluidic channel (~25 to 75% of the total channel height [31]), thereby remaining spatially separated from the electrode interface. Although electric field magnitude decreases with vertical distance from the electrode plane, the confined gap geometry enables sufficient field strength within this cell-trapping region to induce membrane polarization without requiring high global voltages across the full channel height. Because effective electroporation can be achieved at reduced applied voltage, overall current density, Joule heating, and electrochemical reactions at the electrode interface are minimized.

Additionally, the selected 20 µm electrode width balances field localization with fabrication and electrical stability. Maintaining sufficient exposed glass area between electrode features ensures robust PDMS-glass bonding during device assembly and prevents the common bus line from contacting the electrolyte, thereby avoiding unintended short-circuit pathways. This geometric balance supports both reliable electroporation performance and structural integrity during parallelized operation.

The intra-chamber IDE geometry (strip width, gap spacing, and finger number) was intentionally preserved from our previously validated 4 × 10 prototype [27]. The number and lateral distribution of IDE fingers within each chamber were designed to span the footprint of the vortex trapping region. By distributing multiple electrode pairs across the chamber area rather than concentrating them solely at the center, electric field exposure is maintained across both central and peripheral recirculation zones. This configuration helps ensure that cells orbiting along outer vortex trajectories experience sufficient field strength for membrane permeabilization. Informed by prior work [27], inlet and outlet electrode sections connecting to anode and cathode pads were modified for each geometry to maintain electrical performance while accommodating layout-dependent routing.

The overall electrode schematics were converted into equivalent SPICE models representing resistor networks in series and parallel (Figure 2c). Because all chambers operate in parallel and share a common outlet within the integrated microfluidic architecture, experimental measurements reflect aggregate array behavior; chamber-to-chamber uniformity was therefore evaluated through electrical modeling and voltage variability analysis rather than independent chamber isolation. Uniform and efficient electroporation requires both minimal chamber-to-chamber voltage variability and high voltage efficiency, defined as the proportion of input voltage dropped across the array. These metrics were quantified for each configuration, and the input voltage needed to achieve a mean electric field of 900 V/cm (typical for reversible electroporation of mammalian cells [11,32,33]) was determined by SPICE circuit simulation.

Prior experience [27] indicated that minimizing chamber-to-chamber variability benefits from bifurcated inlet and outlet routing pathways that equalize resistance across the array (Figure 2d). This approach was first applied to the VTX-1-style 16 × 12 chamber layout [29]. Although this geometry maximized total chamber count, SPICE simulations revealed substantial resistive losses along extended routing lines created by 12 electrodes in series. Generating a 900 V/cm mean electric field required 39.1 V (voltage efficiency of 48.8 ± 0.87%) and produced 7.70% voltage variability, indicating inefficient and inconsistent voltage transfer (Figure 2f).

Shortening the series path yielded the 16 × 9 configuration, which reduced the required input voltage to 32.2 V and correspondingly increased voltage efficiency to 53.4 ± 0.80%, while lowering chamber-to-chamber variability to 4.51% (Figure 2f). However, even the reduced 32.2 V required for the 16 × 9 layout still exceeded the structural tolerance of the micropatterned electrode traces. Elevated local current densities induce electrochemical erosion, producing voids and ultimately compromising device stability [34,35] (Figure S1).

An alternative 12 × 12 configuration incorporating shorter routing paths and widened inlet/outlet traces was evaluated (Figure 2e). This layout reduced total routing-path resistance by 2.5-fold relative to the 16 × 9 geometry, allowing a greater fraction of input voltage to drop across the array. Achieving a 900 V/cm field required only 24.0 V (Figure 2f), and voltage efficiency increased to 79.4 ± 2.28%. Although chamber-to-chamber variability rose slightly to 11.1%, the reduction in required input voltage mitigated voltage-dependent resistive losses and improved device stability under scaled operating conditions. The degradation observed in the intermediate 16 × 9 configuration was therefore attributed to elevated voltage-induced current density rather than cumulative device usage.

Because this redesign altered the array geometry and routing architecture, its impact on trapping performance was next evaluated. Benchmarking against the commercially validated VTX-1 (16 × 12) configuration [29,30] enabled direct comparison of flow capacity under electroporation-integrated conditions, where additional electrical and material constraints limit achievable flow rates. The VTX-1 system reached flow rates of 8 mL/min (*Re* = 170.6) using a multi-syringe pump, whereas the electroporation-integrated 12 × 12 device was constrained to 5.2 mL/min (*Re* = 140.1) due to PDMS delamination at the micropatterned Au-glass interface. As expected from the reduced chamber count (192 to 144), the overall trapping efficiency of the 12 × 12 device was lower than that of VTX-1 (Figure 2g). When distributed across chambers, the average occupancy per chamber was consistent with prior designs, supporting preservation of size-selective trapping behavior (Figure S2). This maintained trapping performance, together with the substantially improved electrical efficiency and device stability, supported selection of the 12 × 12 layout architecture for downstream electroporation experiments at higher sample volumes within the integrated workflow.

Because the vortex trapping geometry and channel height are unchanged from previously validated heterogeneous spike-in enrichment studies [28,29], and per-chamber trapping efficiency remains comparable following array scaling, these results support preservation of enrichment functionality under integrated electroporation conditions.

Overall, the 12 × 12 configuration provided the most favorable balance between electric field generation, resistive performance, and operational robustness. Final schematic and resistive networks for the 12 × 12 array are provided in Figure S3, and this configuration was selected for all downstream electroporation experiments.

### 3.2. Integrated Trapping–Electroporation Performance Characterization

The feasibility of vortex-assisted electroporation has been demonstrated previously, including with our earlier 4 × 10 platform, which enabled efficient multi-cargo delivery through size-selective pre-concentrating and real-time control of electrical parameters [27,28]. Building on this foundation, the redesigned 12 × 12 array was evaluated using MCF7 cells, a model cell line previously used to assess both vortex trapping and microscale electroporation [27,29], to determine whether integrated trapping and electroporation performance is preserved under scaled operating conditions with increased processing capacity.

To explicitly characterize the platform’s performance at each stage of the microfluidic workflow, we defined four key metrics: trapping efficiency, recovery, viability, and electroporation efficiency. Trapping efficiency (%) was calculated as the number of cells retained within vortex chambers divided by the total number of input cells. Recovery (%) was defined as the number of cells collected at the device outlet following electroporation and wash steps divided by the number of trapped cells. Viability (%) was calculated as the number of live cells divided by the total number of recovered cells. Electroporation efficiency (%) was defined as the number of reporter-positive cells divided by the total number of viable recovered cells. This hierarchical definition allows separation of hydrodynamic performance (trapping and recovery) from biological performance (viability and electroporation), ensuring that delivery outcomes are interpreted relative to the recovered cell population rather than total input.

**Figure 3:**
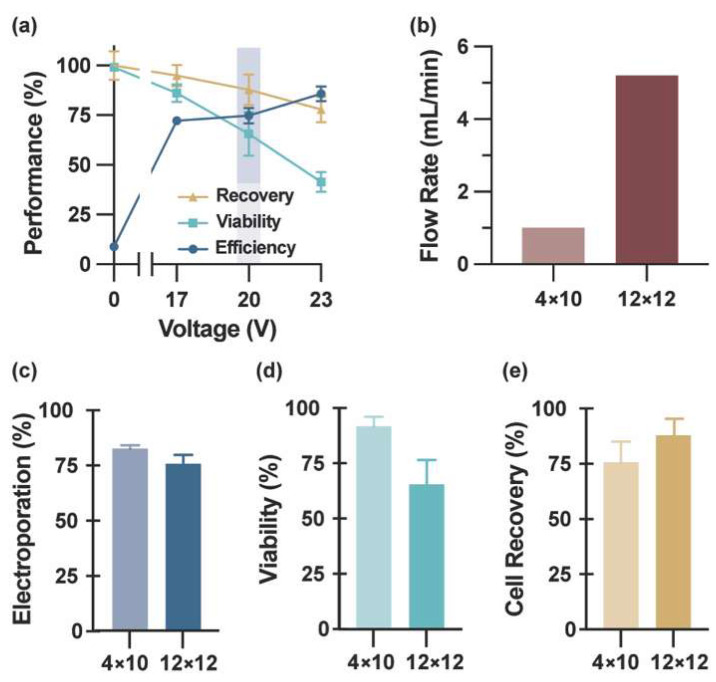
Electroporation performance and processing capacity scaling. (**a**) Electroporation performance of MCF-7 cells quantified by membrane-impermeable dye uptake with post-electroporation viability counterstaining. The gating strategy for identifying successfully electroporated cells is shown in Figure S4. Nuclear labeling performed prior to processing enabled accurate cell enumeration. The shaded gray region denotes the optimal operating voltage range. Data are shown as mean ± SEM (*n* = 3; >300 cells per replicate). (**b**) Sample processing capacity comparison between 4 × 10 and 12 × 12 chamber configurations operated at 40 psi, demonstrating a >5-fold increase in processing capacity with array scaling. Quantitative comparison of (**c**) electroporation efficiency, (**d**) cell viability, and (**e**) cell recovery between 4 × 10 and 12 × 12 devices under optimized conditions. Data are presented as mean ± SEM (*n* = 3).

Electroporation efficiency and viability quantified using YOYO-1 and Calcein AM Red revealed the expected trade-off between membrane permeabilization and cell integrity: increasing input voltage enhanced delivery efficiency but also intensified membrane damage and electrochemical stress, resulting in reduced viability and overall cell recovery (Figure 3a). The highest electroporation efficiency observed was 85.9% at 23 V, accompanied by reduced viability (41.5%) and recovery (78.0%), respectively. Consistent with our prior experience using the lower-capacity 4 × 10 vortex-assisted electroporator [27,28], a modest reduction in transfection efficiency was necessary to achieve practical levels of viability and recovery. Accordingly, 20 V provided the most balanced operating condition, yielding 74.9% delivery efficiency, 65.7% viability, and 88.0% recovery (Figure 3a, blue shading). While we noticed bubble formation with higher voltages (20 V+), the use of high-frequency bipolar pulses (10 kHz) effectively mitigates electrochemical polarization and gas generation at the electrode interface as much as possible. Furthermore, the continuous flow throughout the device aids in dissipating any nucleating bubbles. These results illustrate the characteristic tradeoff between delivery efficiency and cell health in electroporation systems.

Parallelization of the electrode array increased processing capacity while preserving the Reynolds number required for stable vortex trapping in each row. Under these conditions, integration of the solution-exchange system enabled sustained operation at 5.2 mL/min, representing a five-fold increase over the 1.0 mL/min flow capacity of the 4 × 10 platform (Figure 3b). In terms of cellular throughput for molecular delivery experiments, this equates to processing approximately ~2700 cells (based on a standard input concentration of 1 × 10^3^ cells/mL). Given the device’s trapping efficiency (~15%), this yields a capture rate of ~400 cells per standard 4 mL experimental batch. This retention capacity ensures that a sufficient population (>300 cells) is available for statistical analysis in each electroporation event.

Despite this increase in processing capacity, the 12 × 12 array maintained electroporation performance comparable to the 4 × 10 system across delivery efficiency and viability metrics (Figure 3c–e), demonstrating that parallelization enhances processing capacity without substantially compromising delivery outcomes. These observations indicate that the redesigned 12 × 12 array preserves effective vortex trapping and electroporation performance under scaled operation conditions, thereby establishing a stable foundation for subsequent evaluation in primary human cells.

Although the device is intended for single-use operation in primary cell applications to minimize cross-contamination and ensure reproducibility, repeated electroporation experiments were conducted during immortalized cell testing. Stable trapping efficiency, voltage stability, and electroporation performance were maintained over up to 10 consecutive runs under standard operating conditions, indicating practical operational robustness in research use.

### 3.3. Optimization of Electroporation Parameters for Primary Human Cells

Vortex-assisted electroporation has demonstrated robust performance in immortalized cell lines [25,27,28], but extension to human primary cells requires additional optimization given their fragile membranes, limited proliferative capacity, and heightened sensitivity to stress [3]. Having established preserved trapping and delivery performance under scaled operating conditions (Section 3.2), we next evaluated whether the integrated platform could be tuned to support effective gene delivery in primary human cells. We examined whether coordinated electrical and chemical parameter tuning could enable effective transfection in primary cells, using human mammary fibroblasts (HMFs) to define operating conditions that support membrane permeabilization while maintaining recovery and viability within the integrated workflow.

**Figure 4:**
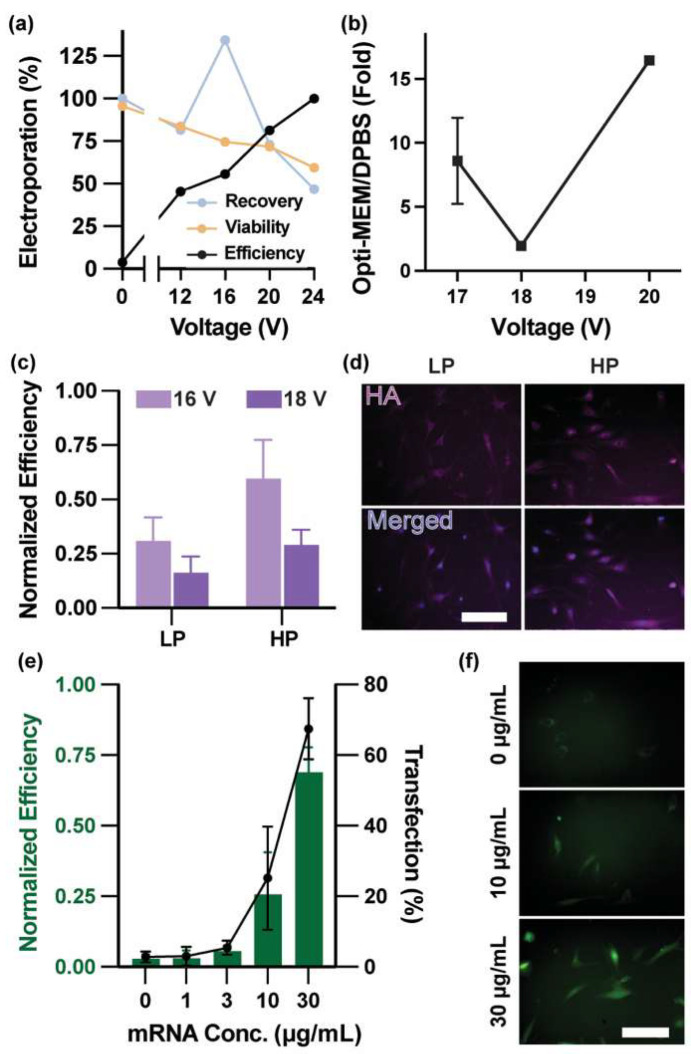
Electrical and Chemical Optimization Enables Multimodal Gene Delivery in Primary Cells. (**a**) Electroporation performance of human mammary fibroblasts (HMFs) in DPBS as a function of applied voltage, quantified by delivery efficiency, viability, and recovery using membrane-impermeable YOYO-1 uptake. (**b**) Fold change in 50 µg/mL ZsGreen plasmid transfection efficiency using Opti-MEM + DMSO relative to DPBS + DMSO across voltages, a voltage-dependent enhancement, with Opti-MEM + DMSO consistently outperforming DPBS + DMSO across the tested voltage range. (**c**) hTERT plasmid transfection efficiency in low-passage (LP, PD < 14) and high-passage (HP, PD > 14) HMFs at 16 V and 18 V, normalized relative to Lipofectamine-mediated delivery. Cells were electroporated in the Opti-MEM + DMSO with 200 µg/mL plasmid and immunostained 48 h post-electroporation. (**d**) Immunofluorescence imaging of HA-tagged hTERT expression in LP and HP HMFs, showing DAPI nuclear staining, HA immunostaining, and merged channels. Scale bars = 100 μm. (**e**) Dose–response analysis of eGFP mRNA electroporation in HMFs. The left *y*-axis shows normalized efficiency relative to an mRNA Lipofectamine control (Messenger Max, 0.1 µg/well), and the right *y*-axis shows absolute transfection efficiency. Data are presented as mean ± SEM (*n* = 3). (**f**) Representative fluorescence microscopy images showing dose-dependent eGFP expression following mRNA electroporation (0, 10, 30 μg/mL). Scale bar = 100 μm.

Quantification of electroporation efficiency, viability, and cell recovery using membrane-impermeable YOYO-1 uptake (Figure 4a) revealed trends consistent with immortalized cell lines: increasing input voltage enhanced membrane permeabilization and delivery efficiency but concurrently produced voltage-dependent reductions in viability and recovery. A balanced operating regime emerged at intermediate voltages (16–20 V), where delivery efficiency increased substantially while maintaining acceptable levels of cell health, establishing electrical conditions for subsequent plasmid delivery studies on the scaled platform.

Optimization efforts were then extended beyond electrical tuning alone using Dulbecco’s Phosphate-Buffered Saline (DPBS) as the electroporation buffer with 50 µg/mL ZsGreen plasmid (4.7 kb). While DPBS supported ZsGreen delivery into HEK 293 cells with 37.5% efficiency (normalized efficiency 0.617; Figure S6), primary human mammary fibroblasts (HMFs) exhibited minimal transfection (0.25%; normalized efficiency of 0.019, Figure S6), even after extensive tuning of voltage, pulse width, pulse counts, and waveform (Figure S7). These results indicate that electrical-parameter optimization alone was insufficient to enable effective transfection in primary cells, motivating investigation of chemical parameter modulation in addition to electrical tuning.

To maintain accessibility, reproducibility, and compatibility with standard laboratory workflows, we intentionally avoided device-specific proprietary electroporation buffers with undisclosed compositions. Many bulk electroporation systems rely on such specialized reagents, which can limit mechanistic insight and complicate integration within microfluidic platforms. Instead, we focused on widely available laboratory media and defined additives to evaluate whether effective primary cell delivery could be achieved under transparent and broadly adoptable conditions.

The EP buffer composition is known to strongly influence membrane permeabilization and cell survival [4,36,37]. We therefore systematically evaluated defined buffer components for primary cell transfection, beginning with dimethyl sulfoxide (DMSO), a membrane-modifying agent [38] known to stabilize hydrophilic pores and lower the electric field threshold for permeabilization [39,40,41]. However, supplementing DPBS with 1% (*v*/*v*) DMSO produced negligible improvement in HMF transfection (Figure S8a), indicating that DPBS-based formulations were inadequate even in the presence of a membrane-permeabilizing additive.

Given the limitation of DPBS, Opti-MEM^TM^, a reduced-serum, sodium bicarbonate-buffered medium reported to support cell viability during electroporation [5,42], was next evaluated as an alternative base buffer. Direct comparison of Opti-MEM + DMSO versus DPBS + DMSO revealed a pronounced voltage-dependent enhancement in transfection efficiency, with Opti-MEM providing the greatest improvement at higher voltage and reduced gains at lower voltages (Figure 4b).

The improved performance observed with the Opti-MEM + DMSO formulation likely reflects combined physicochemical effects. Buffer conductivity influences the effective electric field distribution across the inter-electrode gap and modulates induced transmembrane potential [15]. Differences in osmolarity may alter membrane tension and transient pore stability [43], thereby affecting cargo entry and post-pulse resealing dynamics. The reduced-serum composition of Opti-MEM may further mitigate post-electroporation stress and enhance recovery of primary cells. To facilitate comparison across conditions, all results were normalized to our baseline of 1% (*v*/*v*) DMSO in DPBS at each formulation’s optimal voltage. Using the DMSO-Opti-MEM buffer, we then systematically assessed the combined effects of voltage and plasmid concentration on HMF transfection within the integrated device.

Guided by the YOYO-1 permeabilization data indicating comparable membrane accessibility at intermediate voltages, conditions were grouped into low (<18 V) and high (≥18 V) voltage regimes. Increasing plasmid concentration consistently enhanced delivery, with the highest efficiency achieved at 200 μg/mL plasmid under high-voltage conditions, corresponding to a 46.5-fold improvement over baseline and reaching 88% of Lipofectamine performance (Figure S8c). Fold change was calculated as the ratio of transfection efficiency under the indicated condition to that obtained under the baseline buffer condition (1% DMSO in DPBS at its optimal voltage). However, higher voltages accompanied by reduced downstream cell recovery (Figure S8d), highlighting the inherent tradeoff between maximizing delivery efficiency and preserving viable primary cells. Within this framework, a plasmid concentration of 200 µg/mL combined with intermediate applied voltage (16–18 V) provided the most balanced performance for HMF transfection, excluding 20V due to reduced recovery.

Efficient primary cell electroporation requires coordinated optimization of electrical and chemical parameters. The DMSO-Opti-MEM formulation substantially improved delivery in HMFs, demonstrating that the scaled integrated platform can be tuned to support sensitive primary cells. Transfection requirements vary widely across primary cell types. Unlike bulk electroporation systems that rely on a single static buffer condition, the integrated multi-inlet flow control scheme enables dynamic buffer exchange and real-time modulation of cargo concentration within the sample trapping–electroporation workflow [25,28]. This capability, previously demonstrated in the 4 × 10 prototype for sequential multi-molecule delivery [27,28], allows systematic exploration of formulation parameters without removing cells from the device. Such *in situ* control provides a foundation for automated and combinatorial optimization of electroporation conditions across diverse primary cell types.

### 3.4. Cargo and Cellular Factors Governing Primary Cell Transfection

Robust electroporation performance across diverse applications requires understanding how both cargo characteristics and cellular state influence delivery outcome. Building on the reporter-based validation above, we next extended our analysis to a large, non-reporter plasmid cargo: a hemagglutinin (HA)-tagged human telomerase reverse transcriptase (hTERT) construct (9.0 kb) delivered into HMF cells using the scaled integrated platform. hTERT, the catalytic subunit of the telomerase complex, is tightly regulated in primary somatic cells [44,45,46]. Given that cellular aging alters fibroblast membrane properties and gene expression [6,47,48], electroporation outcomes were compared between low-passage (population doubling (PD) < 14) and high-passage (PD 14–30) HMFs, which differ in proliferative activity as confirmed by Ki67 staining (Figure S9).

Transfection efficiencies of 4.07% (low-passage) and 7.84% (high-passage) were observed at 16 V (E = 960 V/cm), while increasing the voltage to 18 V (E = 1080 V/cm) reduced expression to 2.15% and 3.82%, respectively, indicating non-monotonic voltage dependence and diminished delivery performance at higher field strengths (Figure 4c,d). Higher-passage HMF cells consistently exhibited greater hTERT expression, suggesting that senescence-associated changes may enhance susceptibility to electroporation. Overall transfection efficiencies were lower than those observed with ZsGreen (4.7 kb), likely reflecting the larger size of the hTERT plasmid (9.0 kb). This size-dependent decrease is consistent with prior reports showing reduced delivery efficiency for larger plasmids (6–16 kb) due to increased membrane disruption requirements and slower post-electroporation recovery [49]. These results demonstrate that the platform enables delivery and expression of large, non-reporter genetic cargo, and that delivery efficiency depends jointly on plasmid size and cellular state—important considerations for tailoring electroporation protocols to diverse primary cell types within an integrated trapping–delivery workflow.

To assess platform versatility beyond plasmid DNA, we examined mRNA electroporation, which bypasses nuclear import and transcription and enables rapid protein expression [50]. As chemically modified mRNA can improve translation efficiency and reduce innate immune activation [51,52], we synthesized three distinct eGFP mRNA constructs with different cap and nucleoside chemistries and identified an N1-methylpseudouridine (N1MePsU)-substituted, CleanCap AG-capped variant as the highest-performing candidate (Figure S10). Electroporation of this optimized construct across mRNA concentrations ranging from 0 to 30 µg/mL yielded a maximum expression efficiency of 76.2% at 18 V (E = 1080 V/cm), corresponding to 78% of the efficiency achieved using Lipofectamine (Figure 4e,f). When combined with measured trapping, recovery, and viability fractions, processing of a standard 4 mL sample (1000 cells/mL input concentration) yields an estimated ~170 viable mRNA-transfected primary fibroblasts per experimental run under optimized conditions. Lower mRNA doses further reduce cytotoxicity, highlighting an advantage of mRNA over plasmid DNA for primary cell applications in sensitive cell populations.

These results indicate that the lower delivery efficiencies observed for plasmid cargos (ZsGreen and hTERT), relative to mRNA, arise from cargo-specific limitations, such as size, nuclear import, and transcriptional dependence, rather than constraints of the electroporation platform itself. In contrast, mRNA (~900 nt) bypasses these barriers, enabling rapid and efficient protein expression with reduced cytotoxic burden. Together, these findings demonstrate that the integrated platform supports delivery of diverse nucleic acid cargoes and that delivery outcomes are strongly governed by cargo biology and cellular state. This performance positions the platform for multimodal genetic delivery applications in primary cells processed within a size-selective microfluidic workflow.

## 4. Discussion and Conclusions

In this study, we developed a scaled vortex-integrated electroporation platform for primary cell transfection by integrating size-selective cell trapping, on-chip solution exchange, and systematic optimization of electrical and chemical parameters. Redesigning the electrode array architecture enabled a five-fold increase in processing capacity, while preserving real-time tunability, carrier-free operation, and compatibility with sensitive cell types. The selected 12 × 12 array improved voltage efficiency and maintained stable performance at reduced input voltage.

Sequential optimization of electroporation parameters revealed that both buffer composition and cargo-specific properties critically govern delivery efficiency. An optimized Opti-MEM/DMSO formulation enabled efficient plasmid transfection in human mammary fibroblasts, and further tuning yielded efficiency up to 88% and 78% of Lipofectamine benchmarks for DNA and mRNA delivery, respectively, which generally rely on pre-selected, homogeneous cell populations. The platform successfully delivered both reporter and functional genes, including large plasmids such as hTERT, and uncovered passage-dependent differences in primary cell transfection. Complementary mRNA studies highlighted the role of cargo chemistry, molecular size, and electric field strength in dictating efficiency–viability tradeoffs.

Although plasmid transfection efficiencies in primary HMFs were lower than those observed in immortalized cell lines and required elevated DNA concentrations, these outcomes are consistent with well-documented biological constraints associated with large plasmid delivery into non-immortalized cells, including increased cytotoxic burden and reduced electrotransfer efficiency for larger constructs [49], heightened sensitivity of primary cells to electroporation-induced stress [3,4], and intracellular trafficking barriers such as limited nuclear import and transcriptional dependence [11,14,50]. The use of plasmid concentrations up to 200 µg/mL was intentionally employed to define the upper performance envelope of the integrated system and to characterize the trade space between delivery efficiency and cell recovery. Importantly, mRNA delivery achieved substantially higher efficiencies at lower cytotoxic burden, approaching chemical transfection benchmarks. These findings suggest that electrical field generation and membrane permeabilization within the device are not the primary limiting factors; rather, delivery outcomes are strongly influenced by cargo size and intracellular processing requirements, consistent with prior reports on primary cell electroporation.

In addition to plasmid DNA and mRNA delivery, direct protein and ribonucleoprotein (RNP) delivery represent important strategies for genome editing applications. In our previously reported vortex-assisted electroporation platforms, sequential multi-molecule delivery, including protein cargos, was demonstrated using the same interdigitated electrode chamber geometry [27]. Because the present work preserves the local electroporation configuration, the platform design remains consistent with previously demonstrated protein delivery capabilities and may be extended to RNP delivery. Future studies will evaluate CRISPR-Cas9 RNP delivery within the integrated trapping–electroporation workflow to further expand the platform’s applicability in primary cell engineering.

Direct benchmarking against bulk electroporation systems was not repeated in this study, as the comparative performance of the underlying vortex-assisted electroporation architecture has been previously characterized [26,27,28]. The present work instead focuses on architectural scaling, electrical optimization, and extension to primary human cells within an integrated trapping–delivery workflow. Because bulk electroporation systems typically operate on pre-purified, homogeneous suspensions, comparison based solely on absolute transfection efficiency does not fully reflect the integrated selective trapping and *in situ* parameter control enabled by the microfluidic platform. The preservation of size-selective trapping behavior following electrode array scaling supports compatibility of the integrated architecture with previously demonstrated heterogeneous enrichment workflows.

To contextualize the present platform within the broader landscape of microfluidic electroporation technologies demonstrated in primary cells, representative systems are summarized in Table 1.

As shown in Table 1, while several microfluidic electroporation platforms have demonstrated efficient intracellular delivery, including in certain primary cell types, most operate on pre-purified, homogeneous suspensions and focus exclusively on delivery. In contrast, the present work uniquely integrates size-selective enrichment, parallelized localized electroporation, and on-chip buffer exchange within a unified microfluidic architecture, enabling selective genetic manipulation of heterogeneous primary cell populations.

Several aspects of this work present opportunities for further refinement. Validation was limited to a defined set of cell types, the current assessment emphasized short-term expression rather than long-term functionality, and processing capacity remains bounded by material constraints and modest chamber-to-chamber variability. Ongoing and future efforts will extend evaluation across additional primary cells, incorporate functional cargos, and leverage the multi-inlet architecture for in situ combinatorial buffer screening. Parallel advances in materials and system design will target improved stability and increased parallelization, while longitudinal assays will assess phenotypic durability.

Our work establishes a versatile and scalable electroporation platform for primary cells, enabling efficient delivery of diverse nucleic acid cargos and real-time optimization of delivery conditions. The integrated fluidic architecture provides a foundation for automated, multimodal genetic engineering workflows that bridge selective cell handling and intracellular delivery in a unified system.

## Figures and Tables

**Table 1:** Architectural and Functional Comparison of Representative Microfluidic Electroporation Platforms.

Study	Sorting	Integration	Buffer Exchange	EP Format	Cell Type	Efficiency *	Viability *	Processing Mode
Huang 2017 [21]	None	Delivery	No	Planar (curved)	HUVEC	60–80% (DNA)	>80%	Flow (~1–2 mL/min)
Chang 2020 [17]	None	Delivery	No	Hybrid pulse (flow)	Primary T cells	Moderate–High	70–90%	10^6^–10^7^ cells/min
Lissandrello 2020 [18]	None	Delivery	No	Parallel flow	Primary T cells	~95% (mRNA)	>80%	~20 M cells/min
Welch 2023 [22]	None	Delivery	No	RNP flow	Primary T cells	High (RNP)	70–78%	Clinical-scale flow
Duckert 2022 [20]	None	Delivery	No	Microelectrode array	Primary fibroblasts	60–80%	70–90%	Batch
Little 2023 [23]	None	Delivery	No	Droplet EP	Primary T cells	High	High	Droplet batch
This Work	Vortex (size-based)	Enrichment + EP	Yes	IDE array	Primary fibroblasts	76% (mRNA); 4–8% (DNA)	65–80%	Flow (5.2 mL/min)

* reported as defined in the respective publications. Values for this work represent absolute reporter-positive fractions and post-electroporation viability in primary human fibroblasts under optimized conditions.

## Data Availability

The data supporting the findings of this study are available from the corresponding author upon reasonable request.

## Supplementary Materials

The following supporting information can be downloaded at: https://www.mdpi.com/article/10.3390/mi17030359/s1, Figure S1: Electrode degradation under high-voltage electroporation conditions; Figure S2: Diameter distribution of MCF-7 cells at the device inlet and after vortex trapping; Figure S3: Device architecture and electrical modeling schematic; Table S1: Electrical resistance parameters for SPICE modeling of the vortex-assisted electroporation device; Figure S4: Gating strategy for electroporation eKiciency determination; Figure S5: Comparison of conventional transfection eKiciency in immortalized and primary cells; Figure S6: Comparative vortex-assisted electroporation eKiciency in immortalized and primary cells; Figure S7: Systematic electrical parameter optimization demonstrating insuKicient transfection in primary cells; Figure S8 BuKer- and voltage-dependent modulation of plasmid electroporation in primary cells; Figure S9: Ki67 expression in low- and high-passage HMF cells; Figure S10: Comparison of plasmid DNA and mRNA transfection using conventional reagents in primary HMF cells.

## References

[1] Arul M., Roslani A.C., Cheah S.H. (2017). Heterogeneity in cancer cells: Variation in drug response in different primary and secondary colorectal cancer cell lines in vitro. In Vitr. Cell. Dev. Biol.-Animal.

[2] Richter M., Piwocka O., Musielak M., Piotrowski I., Suchorska W.M., Trzeciak T. (2021). From Donor to the Lab: A Fascinating Journey of Primary Cell Lines. Front. Cell Dev. Biol..

[3] Kucharski M., Mrowiec P., Ocłoń E. (2021). Current standards and pitfalls associated with the transfection of primary fibroblast cells. Biotechnol. Prog..

[4] Jordan E.T., Collins M., Terefe J., Ugozzoli L., Rubio T. (2008). Optimizing Electroporation Conditions in Primary and Other Difficult-to-Transfect Cells. J. Biomol. Tech..

[5] Hyder I., Eghbalsaied S., Kues W.A. (2020). Systematic optimization of square-wave electroporation conditions for bovine primary fibroblasts. BMC Mol. Cell Biol..

[6] Macieira-Coelho A., Bourne G.H., Danielli J.F., Jeon K.W. (1983). Changes in Membrane Properties Associated with Cellular Aging. International Review of Cytology.

[7] Thomas C.E., Ehrhardt A., Kay M.A. (2003). Progress and problems with the use of viral vectors for gene therapy. Nat. Rev. Genet..

[8] Li X., Ma Y., Xue Y., Zhang X., Lv L., Quan Q., Chen Y., Yu G., Liang Z., Zhang X. (2023). High-throughput and efficient intracellular delivery method via a vibration-assisted nanoneedle/microfluidic composite system. ACS Nano.

[9] Sharei A., Zoldan J., Adamo A., Sim W.Y., Cho N., Jackson E., Mao S., Schneider S., Han M.-J., Lytton-Jean A. (2013). A vector-free microfluidic platform for intracellular delivery. Proc. Natl. Acad. Sci. USA.

[10] Chakrabarty P., Abinaya R., Suzuki R., Vedantam S., Rao S., Nagai M., Santra T.S. (2025). High throughput intracellular delivery using a 2D cell-squeezing mechanoporation device and its analysis by a deep learning model. Adv. Healthc. Mater..

[11] Rols M.-P., Teissié J. (1998). Electropermeabilization of Mammalian Cells to Macromolecules: Control by Pulse Duration. Biophys. J..

[12] Zhang Z., Qiu S., Zhang X., Chen W. (2018). Optimized DNA electroporation for primary human T cell engineering. BMC Biotechnol..

[13] Wu M.H., Smith S., Danet G., Lin A., Williams S., Liebowitz D., Dolan M. (2001). Optimization of culture conditions to enhance transfection of human CD34^+^ cells by electroporation. Bone Marrow Transplant..

[14] Yarmush M.L., Golberg A., Serša G., Kotnik T., Miklavčič D. (2014). Electroporation-Based Technologies for Medicine: Principles, Applications, and Challenges. Annu. Rev. Biomed. Eng..

[15] Kotnik T., Rems L., Tarek M., Miklavčič D. (2019). Membrane Electroporation and Electropermeabilization: Mechanisms and Models. Annu. Rev. Biophys..

[16] Choi S.-E., Khoo H., Hur S.C. (2022). Recent Advances in Microscale Electroporation. Chem. Rev..

[17] Chang A.-Y., Liu X., Tian H., Hua L., Yang Z., Wang S. (2020). Microfluidic Electroporation Coupling Pulses of Nanoseconds and Milliseconds to Facilitate Rapid Uptake and Enhanced Expression of DNA in Cell Therapy. Sci. Rep..

[18] Lissandrello C.A., Santos J.A., Hsi P., Welch M., Mott V.L., Kim E.S., Chesin J., Haroutunian N.J., Stoddard A.G., Czarnecki A. (2020). High-throughput continuous-flow microfluidic electroporation of mRNA into primary human T cells for applications in cellular therapy manufacturing. Sci. Rep..

[19] VanderBurgh J.A., Corso G.T., Levy S.L., Craighead H.G. (2024). A multiplexed microfluidic continuous-flow electroporation system for efficient cell transfection. Biomed. Microdevices.

[20] Duckert B., Fauvart M., Goos P., Stakenborg T., Lagae L., Braeken D. (2022). High-definition electroporation: Precise and efficient transfection on a microelectrode array. J. Control. Release.

[21] Huang D., Zhao D., Li J., Wu Y., Zhou W., Wang W., Liang Z., Li Z. (2017). High cell viability microfluidic electroporation in a curved channel. Sens. Actuators B Chem..

[22] Welch M., Flusberg D.A., Hsi P., Haroutunian N.J., Santos J.A., Kim E.S., Markovic S., Coppeta J.R., Lissandrello C.A., Balestrini J.L. (2023). High-Throughput CRISPR/Cas9 Mediated Gene Editing of Primary Human T Cells in a Microfluidic Device for Cellular Therapy Manufacturing. Adv. Mater. Technol..

[23] Little S.R., Leung Z., Quach A.B., Hirukawa A., Gholizadeh F., Hajiaghayi M., Darlington P.J., Shih S.C. (2023). A Tri-Droplet Liquid Structure for Highly Efficient Intracellular Delivery in Primary Mammalian Cells Using Digital Microfluidics. Adv. Mater. Technol..

[24] Zhan Y., Wang J., Bao N., Lu C. (2009). Electroporation of Cells in Microfluidic Droplets. Anal. Chem..

[25] Vickers D.A.L., Ouyang M., Choi C.H., Hur S.C. (2014). Direct drug cocktail analyses using microscale vortex-assisted electroporation. Anal. Chem..

[26] Yun H., Hur S.C. (2013). Sequential multi-molecule delivery using vortex-assisted electroporation. Lab Chip.

[27] Ouyang M., Hill W., Lee J.H., Hur S.C. (2017). Microscale symmetrical electroporator array as a versatile molecular delivery system. Sci. Rep..

[28] Sung H.W., Choi S.-E., Chu C.H., Ouyang M., Kalyan S., Scott N., Hur S.C. (2022). Sensitizing drug-resistant cancer cells from blood using microfluidic electroporator. PLoS ONE.

[29] Che J., Yu V., Dhar M., Renier C., Matsumoto M., Heirich K., Garon E.B., Goldman J., Rao J., Sledge G.W. (2016). Classification of large circulating tumor cells isolated with ultra-high throughput microfluidic Vortex technology. Oncotarget.

[30] Lemaire C.A., Liu S.Z., Wilkerson C.L., Ramani V.C., Barzanian N.A., Huang K.-W., Che J., Chiu M.W., Vuppalapaty M., Dimmick A.M. (2018). Fast and Label-Free Isolation of Circulating Tumor Cells from Blood: From a Research Microfluidic Platform to an Automated Fluidic Instrument, VTX-1 Liquid Biopsy System. SLAS Technol..

[31] Hur S.C., Mach A.J., Di Carlo D. (2011). High-throughput size-based rare cell enrichment using microscale vortices. Biomicrofluidics.

[32] Fox M.B., Esveld D.C., Valero A., Lüttge R., Mastwijk H.C., Bartels P.V., Van Den Berg A., Boom R.M. (2006). Electroporation of cells in microfluidic devices: A review. Anal. Bioanal. Chem..

[33] Weaver J.C. (1993). Electroporation: A general phenomenon for manipulating cells and tissues. J. Cell. Biochem..

[34] Black J.R. (1969). Electromigration—A brief survey and some recent results. IEEE Trans. Electron Devices.

[35] Hummel R.E. (1994). Electromigration and related failure mechanisms in integrated circuit interconnects. Int. Mater. Rev..

[36] Sherba J.J., Hogquist S., Lin H., Shan J.W., Shreiber D.I., Zahn J.D. (2020). The effects of electroporation buffer composition on cell viability and electro-transfection efficiency. Sci. Rep..

[37] Alawar N., Schirra C., Hohmann M., Becherer U. (2024). A solution for highly efficient electroporation of primary cytotoxic T lymphocytes. BMC Biotechnol..

[38] Fernández M.L., Reigada R. (2014). Effects of Dimethyl Sulfoxide on Lipid Membrane Electroporation. J. Phys. Chem. B.

[39] Melkonyan H., Sorg C., Klempt M. (1996). Electroporation Efficiency in Mammalian Cells is increased by Dimethyl Sulfoxide (DMSO). Nucleic Acids Res..

[40] Gironi B., Kahveci Z., McGill B., Lechner B.-D., Pagliara S., Metz J., Morresi A., Palombo F., Sassi P., Petrov P.G. (2020). Effect of DMSO on the Mechanical and Structural Properties of Model and Biological Membranes. Biophys. J..

[41] Ménorval M.-A., de Mir L.M., Fernández M.L., Reigada R. (2012). Effects of Dimethyl Sulfoxide in Cholesterol-Containing Lipid Membranes: A Comparative Study of Experiments In Silico and with Cells. PLoS ONE.

[42] Eghbalsaied S., Kues W.A. (2023). An Electrochemical Protocol for CRISPR-Mediated Gene-Editing of Sheep Embryonic Fibroblast Cells. Cells Tissues Organs.

[43] Teissie J., Golzio M., Rols M.P. (2005). Mechanisms of cell membrane electropermeabilization: A minireview of our present (lack of ?) knowledge. Biochim. Biophys. Acta.

[44] Soares J., Lowe M.M., Jarstfer M.B. (2011). The Catalytic Subunit of Human Telomerase Is a Unique Caspase-6 and Caspase-7 Substrate. Biochemistry.

[45] Bryan T.M., Cohen S.B. (2023). Telomerase. Handbook of Chemical Biology of Nucleic Acids.

[46] Tran D.A., Wong T.C., Schep A.N., Drewell R.A. (2010). Characterization of an Ultra-Conserved Putative *cis*-Regulatory Module at the Mammalian Telomerase Reverse Transcriptase Gene. DNA Cell Biol..

[47] Lou P.-J., Chiu M.-Y., Chou C.-C., Liao B.-W., Young T.-H. (2010). The effect of poly (ethylene-co-vinyl alcohol) on senescence-associated alterations of human dermal fibroblasts. Biomaterials.

[48] Chen X., Li Z., Feng Z., Wang J., Ouyang C., Liu W., Fu B., Cai G., Wu C., Wei R. (2006). Integrin-Linked Kinase Induces Both Senescence-Associated Alterations and Extracellular Fibronectin Assembly in Aging Cardiac Fibroblasts. J. Gerontol. Ser. A.

[49] Lesueur L.L., Mir L.M., André F.M. (2016). Overcoming the Specific Toxicity of Large Plasmids Electrotransfer in Primary Cells In Vitro. Mol. Ther. Nucleic Acids.

[50] Youn H., Chung J.-K. (2015). Modified mRNA as an alternative to plasmid DNA (pDNA) for transcript replacement and vaccination therapy. Expert Opin. Biol. Ther..

[51] Parr C.J.C., Wada S., Kotake K., Kameda S., Matsuura S., Sakashita S., Park S., Sugiyama H., Kuang Y., Saito H. (2020). N1-Methylpseudouridine substitution enhances the performance of synthetic mRNA switches in cells. Nucleic Acids Res..

[52] Roy B., McMahon M. (2021). Effects of mRNA Modifications on Translation: An Overview. RNA Modifications: Methods and Protocols.

