# Supplementary material for: Integrated Size-Selective Cell Purification and Electroporation for Genetic Manipulation of Primary Cells
Source: Micromachines (Basel). 2026 Mar 15;17(3):359. doi: 10.3390/mi17030359 (PMC13029108; doi:10.3390/mi17030359)
Supplement: Supplementary file 1 [file micromachines-17-00359-s001.zip › micromachines-4189722-supplementary.pdf]

**Supplementary Materials**

## Integrated Size-Selective Cell Purification and Electroporation for Genetic Manipulation of Primary Cells

Hyun Woo Sung and Soojung Claire Hur

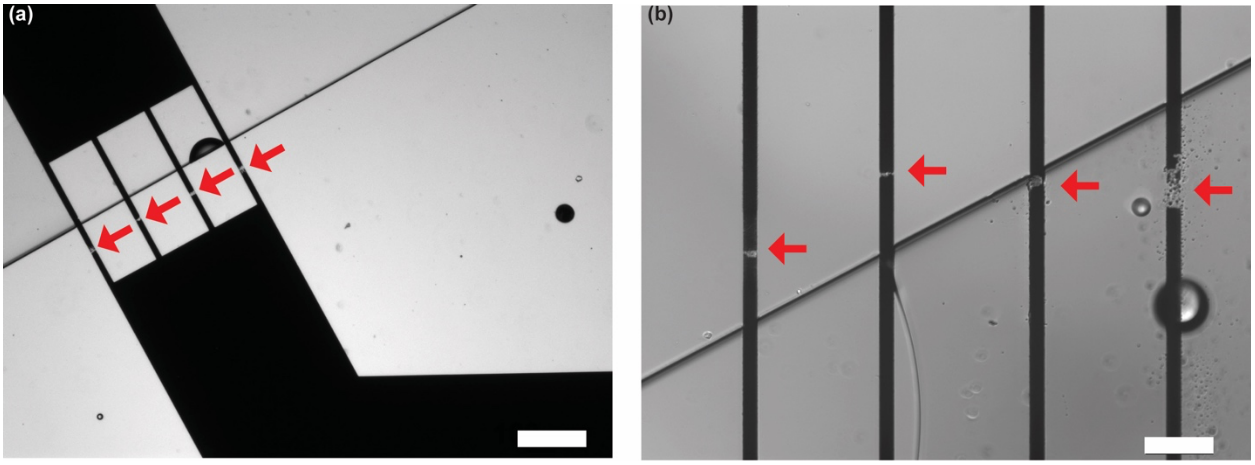


**Figure S1: Electrode degradation under high-voltage electroporation conditions.** (a) Brightfield micrograph showing electrode erosion and void formation (red arrows) along common bus lines within thin electrode regions following high-voltage operation. Degradation is most pronounced in regions of elevated electrical resistance, consistent with localized current crowding and electrochemical damage. Scale bar = 250 µm. (b) Higher-magnification view of representative erosion and void formation sites corresponding to the regions indicated in (a). Scale bar = 100 µm.

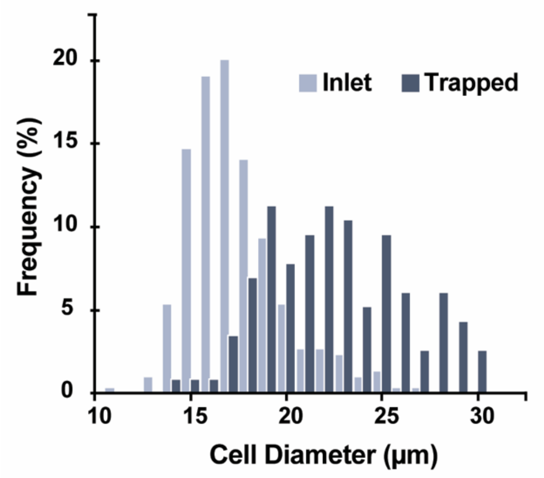


**Figure S2 Diameter distribution of MCF-7 cells at the device inlet and after vortex trapping.** Overlaid histograms show the cell size distribution at the inlet (light gray) and for

vortex-trapped cells (dark gray), revealing selective enrichment of larger cells. Mean cell diameters increased from 16.9 ± 2.5 μm at the inlet to 22.1 ± 3.6 μm after trapping.

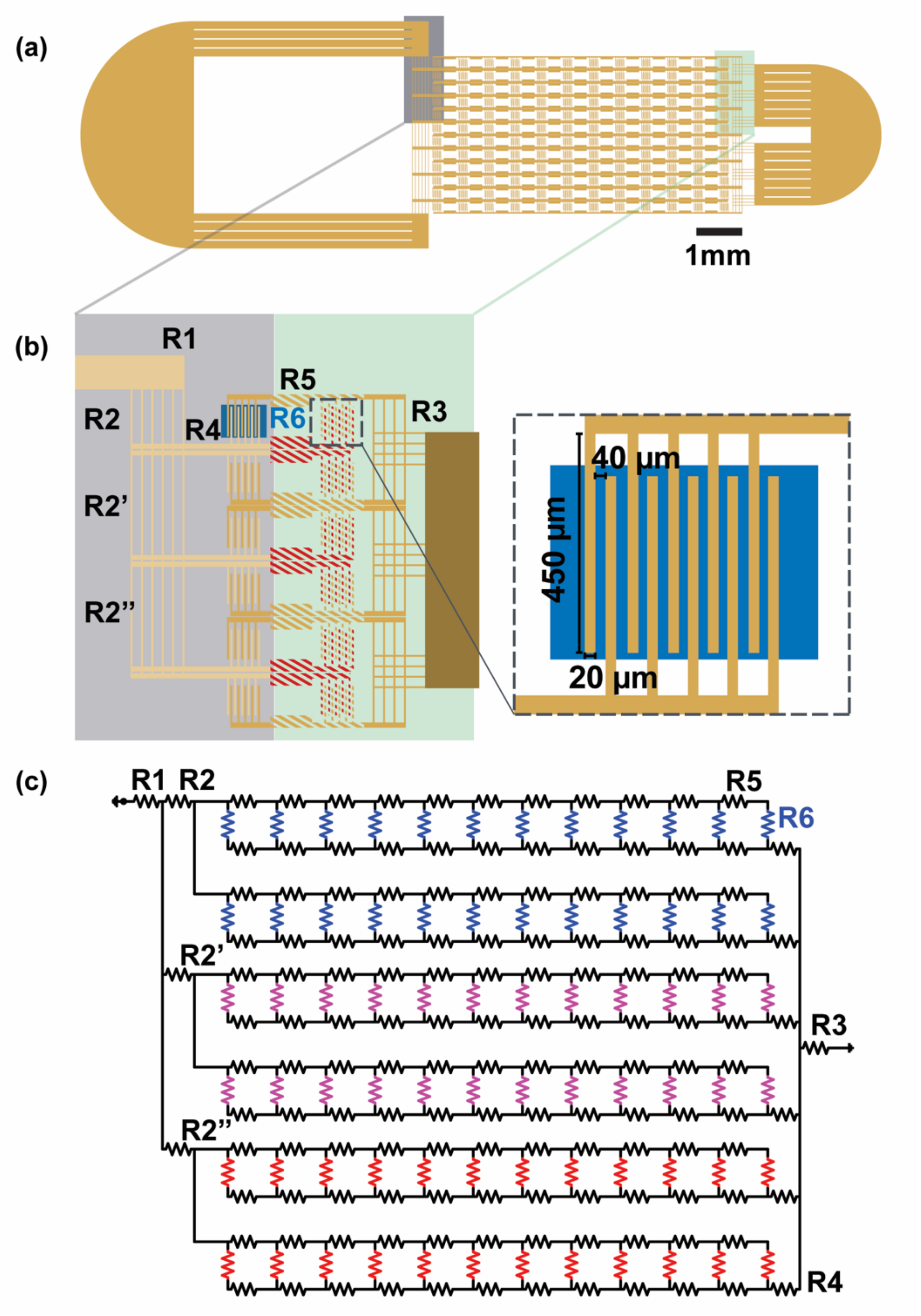


**Figure S3: Device architecture and electrical modeling schematic.** (a) CAD rendering of the patterned Au electrode layout used for vortex-assisted electroporation, showing the overall electrode geometry and routing pathways. The dashed box highlights the electroporation chamber array. Scale bar = 1 mm. (b) Expanded view of the electrode layout

and routing architecture. Labeled resistor elements (R1–R6) correspond to distinct electrode and routing regions used for circuit modeling. Geometric dimensions and resistance values for each region are summarized in **Table S1.** The dashed box shows a magnified view of a single electroporation chamber comprising five pairs of interdigitated electrodes, with key geometric dimensions indicated. (c) Equivalent electrical resistance network used for SPICE modeling of the electrode array. Individual resistors represent geometrically distinct electrode and routing segments, with color coding corresponding to different resistance regions. Only the upper half of the network is shown, as device symmetry results in identical resistive behavior in both halves.

| *Name of Resistor* | *Component* | *Inlet/Outlet* | *Length (µm)* | *Width (µm)* | *CS Area (µm2)* | *R (Ω)* | *# in Parallel* | *Parallel Res. (Ω)* | *Total Resistance* |
|---|---|---|---|---|---|---|---|---|---|
| **R1** | E1 | Inlet | 15440 | 500 | 150 | 2.51 | 4 | 0.63 | **0.63** |
| **R2** | E2 | Inlet | 760 | 20 | 6 | 3.09 | 6 | 0.52 | **0.92** |
| **R2'** | E3 | Inlet | 2484 | 20 | 6 | 10.10 | 6 | 1.68 | **2.09** |
| **R2''** | E4 | Inlet | 4280 | 20 | 6 | 17.41 | 6 | 2.90 | **3.31** |
| | E1 | In-Between | 800 | 80 | 24 | 0.81 | 2 | 0.41 | |
| **R6** | Chamber | In-Between | | | *COMSOL Simulation* | | | | **401.77** |
| **R5** | E1 | Outlet | 920 | 80 | 24 | 0.94 | 1 | 0.94 | |
| | E2 | Outlet | 800 | 180 | 54 | 0.36 | 1 | 0.36 | **1.30** |
| **R4** | E1 | Inlet | 800 | 80 | 24 | 0.81 | 1 | 0.81 | |
| | E2 | Inlet | 451 | 20 | 6 | 1.83 | 4 | 0.46 | **1.27** |
| **R3** | E1 | Outlet | 800 | 20 | 6 | 3.25 | 12 | 0.27 | |
| | E2 | Outlet | 3080 | 500 | 150 | 0.50 | 12 | 0.04 | **0.31** |

| *Constants* | | | |
|---|---|---|---|
| | *Resistivity* | *0.0244* | *[Ω*µm]* |
| | *Thickness* | *0.3* | *µm* |

**Table S1: Electrical resistance parameters for SPICE modeling of the vortex-assisted electroporation device**. Geometric dimensions of electrode and routing regions corresponding to resistors R1–R6 in **Figure S2** were extracted from CAD designs and converted to electrical resistance values using the resistivity of gold (0.0244 Ω·µm) and a deposited electrode thickness of 0.3 µm. Resistance calculations account for segment length, width, cross-sectional area, and parallel circuit configurations. Individual resistances were combined according to circuit topology to determine the effective resistance of each device region. The electroporation chamber resistance (R6) was obtained from COMSOL finite-element simulations. These parameters were used for SPICE-based modeling of voltage distribution and electric field uniformity.

E1–E4 denote electrode regions; CS area, cross-sectional area; # in parallel, number of parallel resistive elements; Parallel Res., equivalent parallel resistance.

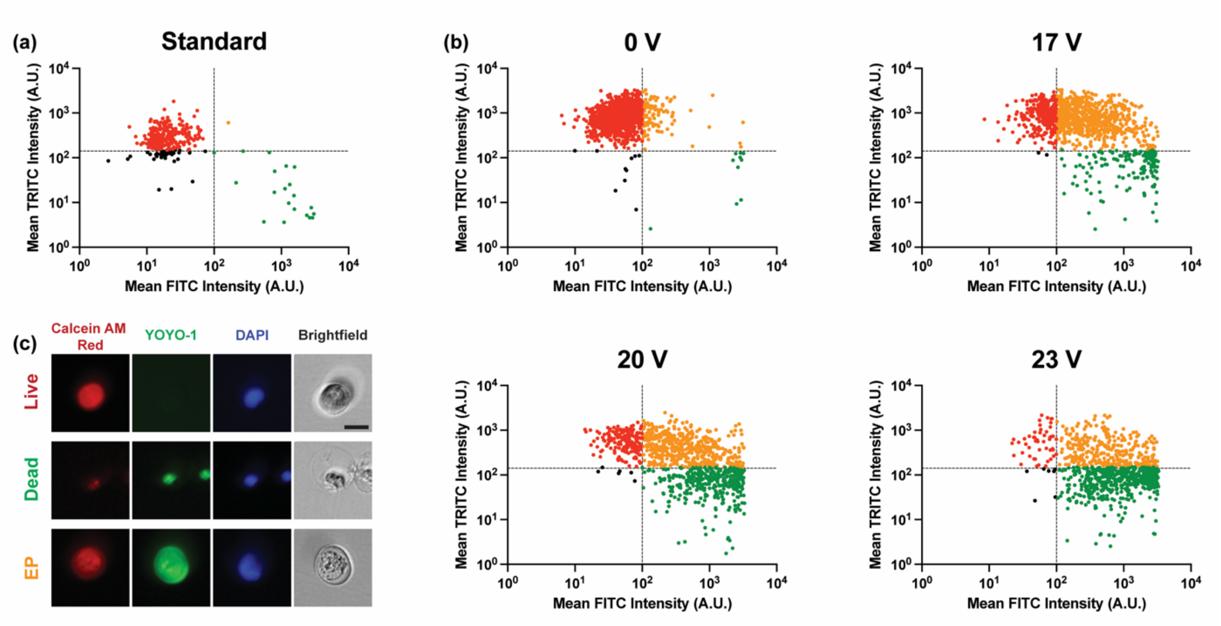


**Figure S4. Gating strategy for electroporation efficiency determination.** (a) Standard control used to establish fluorescence intensity thresholds for cell viability and electroporation status. The viability threshold (Calcein Red-AM, y-axis) was set at the maximum fluorescence intensity of dead cells to minimize false-positive classification. The electroporation threshold (YOYO-1, x-axis) was defined such that fewer than 1% of viable control cells exceeded this value. (b) Application of the standardized gating thresholds to electroporated cell populations across applied voltages (0–23 V). Color-coded subpopulations correspond to viable non-electroporated cells (red), viable electroporated cells (orange), and dead or lysed cells (green) attributed to electroporation-induced membrane damage. Events falling below both viability and electroporation thresholds (black) were excluded from efficiency calculations. Voltage-dependent shifts in population distributions indicate the emergence of optimal electroporation conditions at intermediate voltages. (c) Representative fluorescence and brightfield images of viable, electroporated, and dead cells, showing Calcein Red-AM, YOYO-1, DAPI, and corresponding brightfield channels. Scale bar = 20 μm.

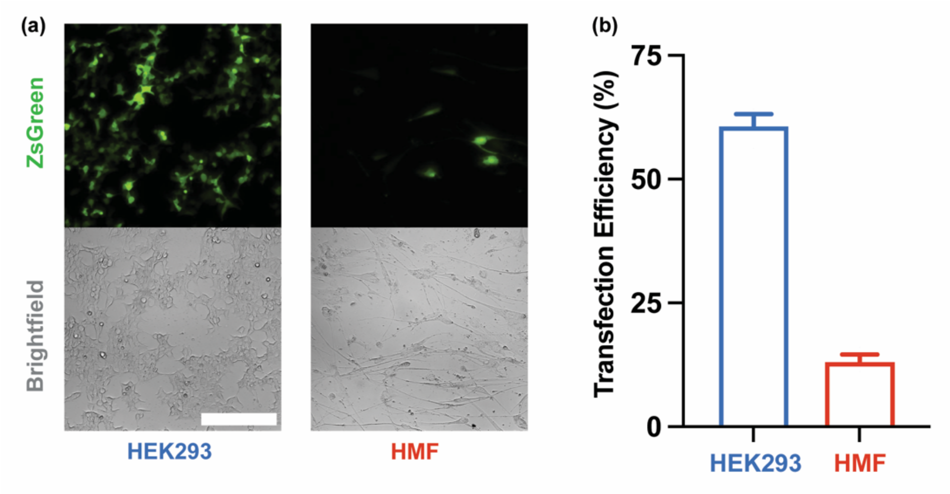


**Figure S5: Comparison of conventional transfection efficiency in immortalized and primary cells.** (a) Representative fluorescence (ZsGreen) and corresponding brightfield microscopy images of HEK293 (immortalized) and human mammary fibroblast (HMF, primary) cells 24 h post-transfection using a conventional chemical transfection reagent. Scale bar = 100 µm. (b) Quantification of transfection efficiency at 24 h post-transfection, calculated as the fraction of ZsGreen-positive cells relative to the total DAPI-positive cell population. Data are presented as mean ± SEM (n = 3; >200 cells per replicate).

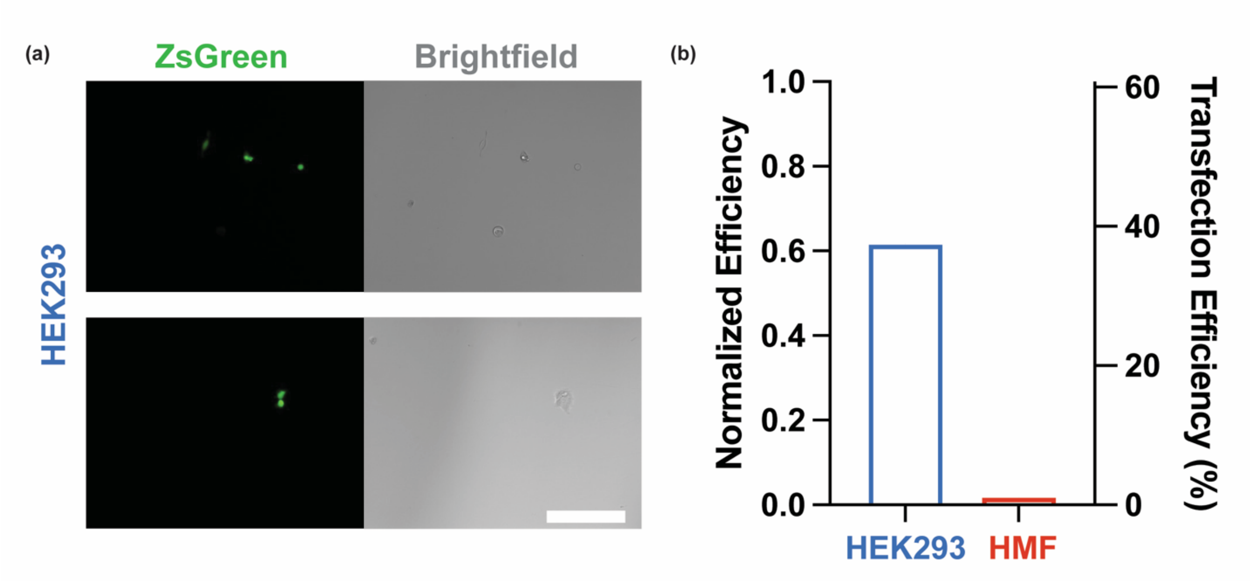


**Figure S6: Comparative vortex-assisted electroporation efficiency in immortalized and primary cells.** (a) Representative fluorescence (ZsGreen) and corresponding brightfield microscopy images of HEK293 (immortalized) cells following device-based electroporation in DPBS base buffer without DMSO (50 µg/mL plasmid; 20 V input, 10 kHz frequency, 20 pulses). Images were acquired 24 h post-electroporation. Lower apparent cell density in

HEK293 samples reflects reduced trapping efficiency associated with smaller cell size. HFM (primary) cells are not shown. Scale bar = 100 μm. (b) Quantitative comparison of electroporation efficiency between HEK293 and HMF cells under identical operating conditions. The left y-axis shows electroporation efficiency normalized to Lipofectamine-mediated transfection efficiency measured for each cell type (Figure S5), enabling cell-type-independent comparison of delivery performance. The right y-axis shows the corresponding observed electroporation transfection efficiency.

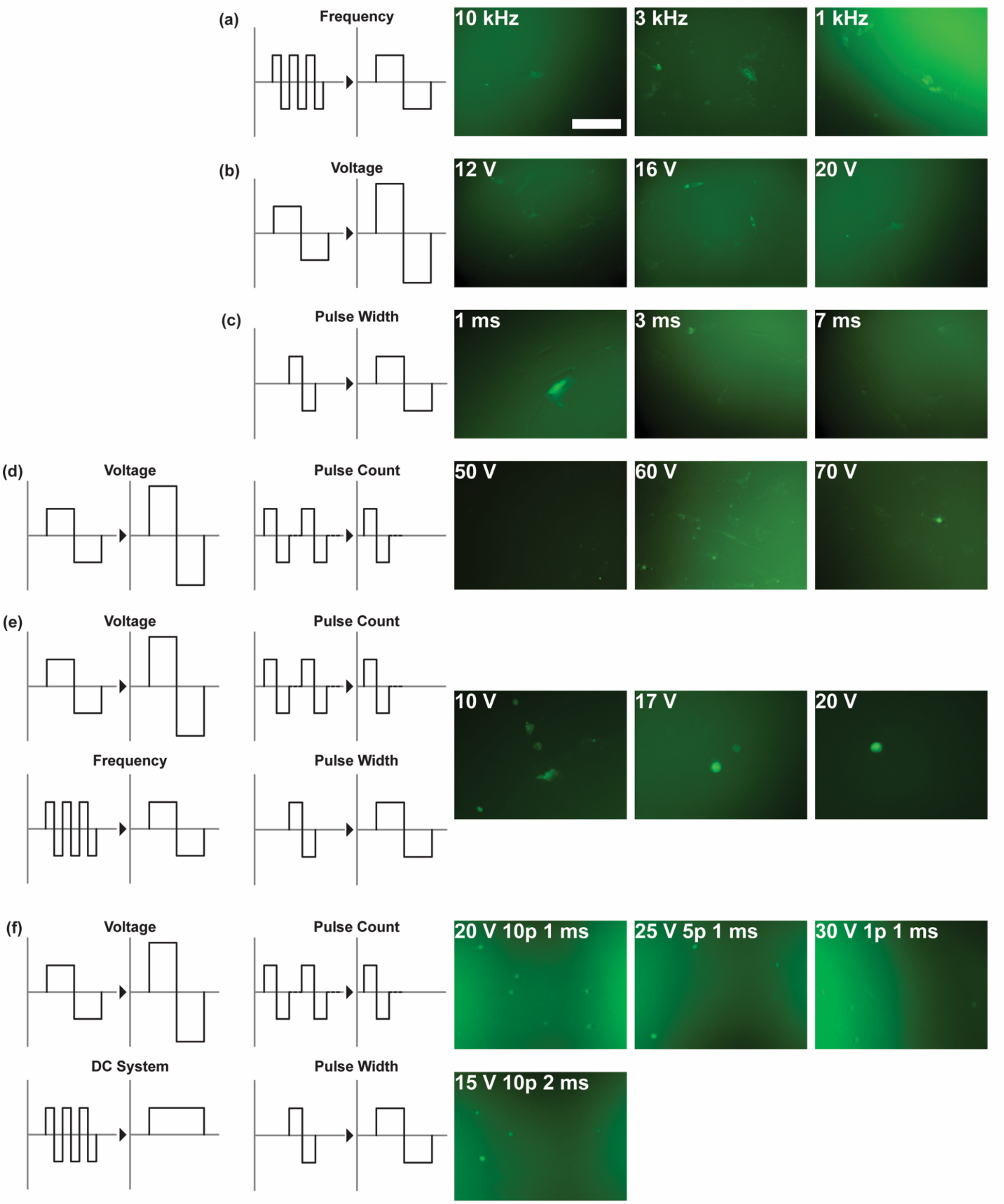


**Figure S7: Systematic electrical parameter optimization demonstrating insufficient transfection in primary cells.** Schematic waveforms (left) illustrate the electrical parameters varied for each condition. Unless otherwise noted, base electroporation parameters consisted of 20 pulses of 1 ms AC square waves at 20 V and 10 kHz, with a 1 s

inter-pulse interval. Fluorescence microscopy images (right) show ZsGreen expression in HMF cells acquired 72 h post-electroporation. Nomenclature: V, voltage; kHz, kilohertz; p, pulse count. (a–c) Single-parameter optimization experiments showing electroporation outcomes with varying (a) pulse frequency (10, 3, and 1 kHz), (b) voltage amplitude (12, 16, and 20 V), and (c) pulse width (1, 3, and 7 ms). (d) Dual-parameter optimization combining elevated voltage amplitudes (50–70 V) with reduced pulse counts (2 pulses) to mitigate electrode erosion. (e) Four-parameter multiplexed optimization integrating voltage, pulse count, frequency, and pulse width. Representative conditions (10, 17, and 20 V) exhibit persistently low fluorescence intensity despite increased waveform complexity. (f) Multiplexed optimization using a DC electroporation system. The top row shows pulsed conditions (20 V, 10 pulses, 1 ms; 25 V, 5 pulses, 1 ms; 30 V, 1 pulse, 1 ms), while the bottom row shows DC conditions (15 V, 100 pulses, 2 ms).

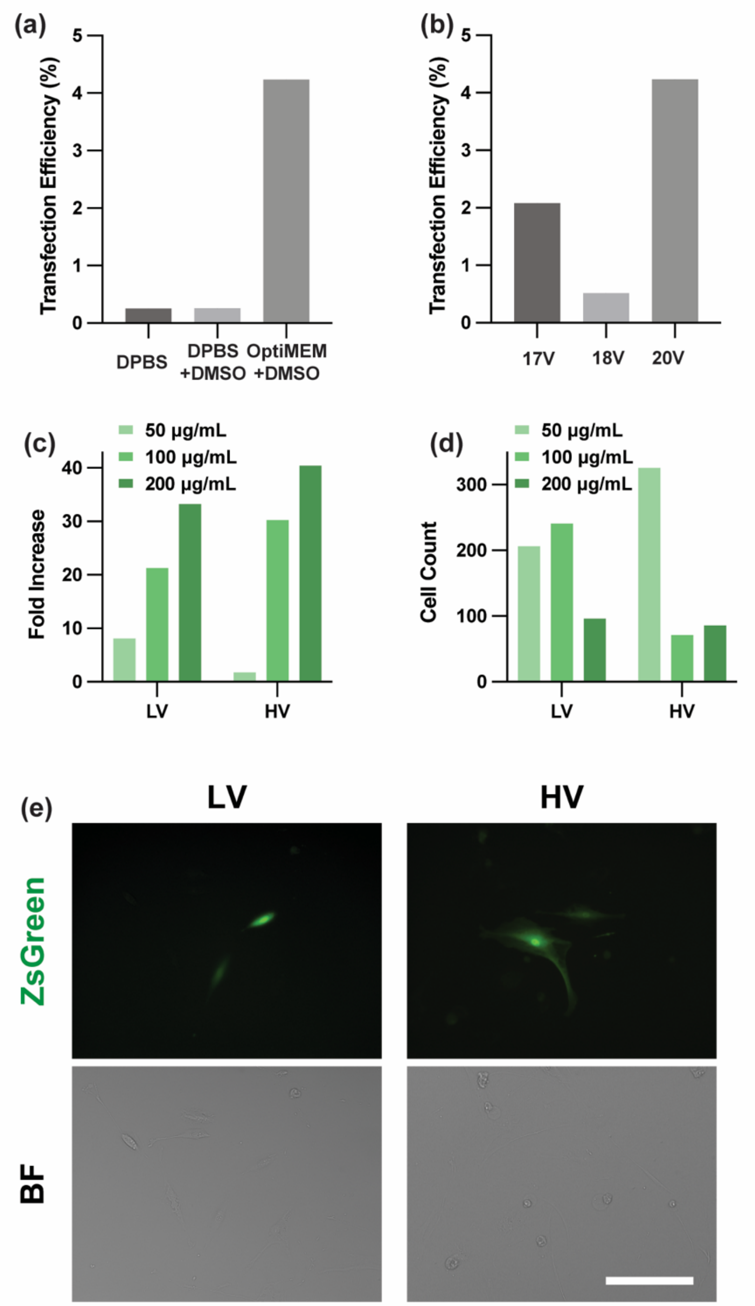


**Figure S8 Buffer- and voltage-dependent modulation of plasmid electroporation in primary cells.** (a) Absolute ZsGreen plasmid transfection efficiency at 20 V using 50 µg/mL plasmid, comparing DPBS, DPBS + DMSO, and Opti-MEM + DMSO buffer formulations. (b) ZsGreen plasmid transfection efficiency as a function of applied voltage (17, 18, and 20 V) using Opti-MEM + DMSO with a fixed plasmid concentration of 50 µg/mL.(c) Fold increase in transfection efficiency relative to the DPBS + DMSO baseline for low-voltage (LV) and high-voltage (HV) regimes across plasmid concentrations (50, 100, and 200 µg/mL). (d) Collected cell counts corresponding to the LV and HV conditions shown in (c), illustrating reduced

recovery at higher voltages. (e) Representative fluorescence (ZsGreen) and corresponding brightfield (BF) microscopy images of HMF cells electroporated under LV and HV conditions using 200 µg/mL plasmid. Increased incidence of burst cells is observed under HV conditions. Scale bar = 100 µm.

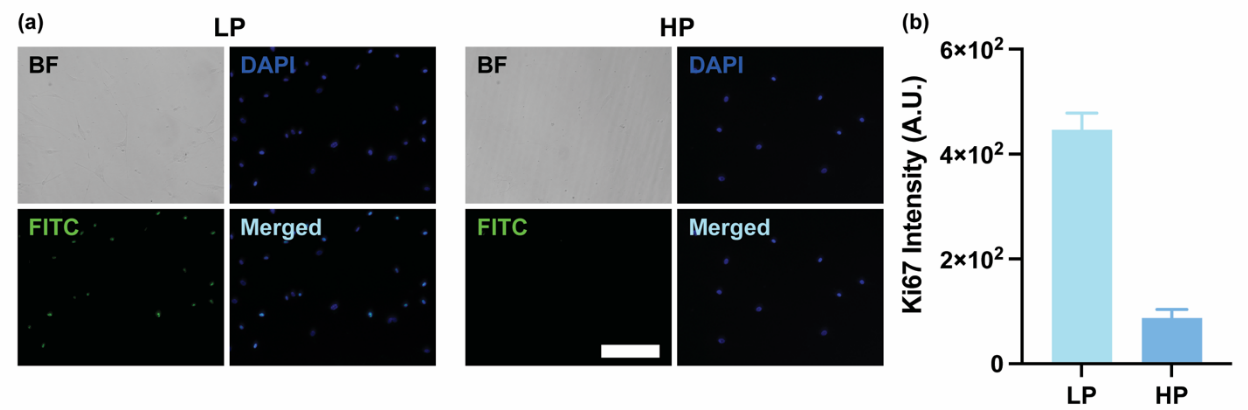


**Figure S9: Ki67 expression in low- and high-passage HMF cells.** (a) Representative brightfield (BF) and fluorescence microscopy images of Ki67 immunostaining in low-passage (LP, left) and high-passage (HP, right) human mammary fibroblast (HMF) cells. Images show DAPI nuclear staining (blue), Ki67 immunofluorescence (FITC, green), and merged channels. Scale bar = 100 µm. (b) Quantitative analysis of Ki67 fluorescence intensity per cell, demonstrating significantly higher proliferative activity in LP compared to HP HMF cells. Error bars represent mean ± SEM (>200 cells per condition).

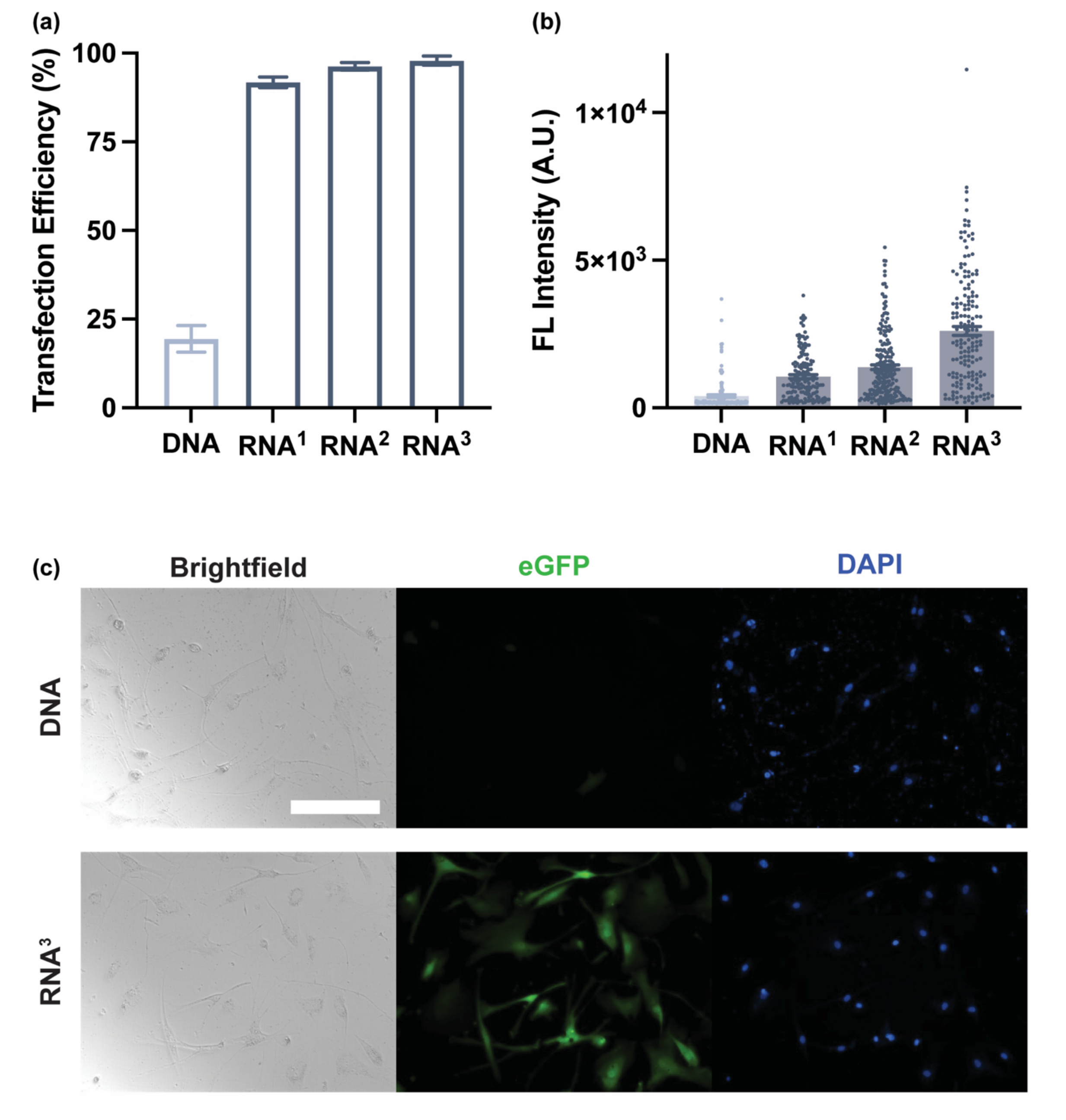


**Figure S10: Comparison of plasmid DNA and mRNA transfection using conventional reagents in primary HMF cells.** (a) Transfection efficiency comparison between eGFP plasmid DNA and three eGFP mRNA variants: $RNA^1$ (ARCA cap, N1-methylpseudouridine), $RNA^2$ (CleanCap AG, 5-methoxyuridine), and $RNA^3$ (CleanCap AG, N1-methylpseudouridine). mRNA nomenclature denotes cap analog and uridine modification, respectively. Error bars represent mean ± SEM (n = 3). (b) Single-cell fluorescence intensity analysis showing differences in expression levels among cargo types despite comparable transfection efficiencies. $RNA^3$ exhibited the highest eGFP expression in HMF cells and was selected for subsequent device-based mRNA electroporation experiments. Error bars represent mean ±

SEM (n > 150 cells). (c) Representative brightfield and fluorescence microscopy images of HMF cells transfected with eGFP plasmid DNA (DNA) or eGFP mRNA (RNA[3]) using cargo-specific conventional transfection reagents. Images show brightfield (left), eGFP fluorescence (center), and DAPI nuclear staining (right). Scale bar = 100 μm.
